# Impact of Respiratory Effort Parameters on Clinical Outcomes in Respiratory Failure Patients (Effort-I): A Prospective Observational Study

**DOI:** 10.1016/j.aicoj.2026.100103

**Published:** 2026-06-20

**Authors:** Phruet Soipetkasem, Touchapong Taksinwarajarn, Detajin Junhasavasdikul, Yuda Sutherasan, Pongdhep Theerawit

**Affiliations:** a Division of Critical Care Medicine Department of Medicine, Faculty of Medicine Ramathibodi Hospital, Mahidol University, Bangkok, Thailand; b Division of Pulmonary and Pulmonary Critical Care Medicine Department of Medicine, Faculty of Medicine Ramathibodi Hospital, Mahidol University, Bangkok, Thailand

**Keywords:** Respiratory effort, Mechanical ventilation, P_0.1_ Transpulmonary driving pressure, Ventilator-free days

## Abstract

**Background:**

Excessive or insufficient respiratory drive and inspiratory effort during mechanical ventilation may worsen outcomes through patient self-inflicted lung injury or diaphragm disuse. We evaluated whether bedside measures of respiratory drive and effort during the first 48 h of ventilation were associated with outcomes in critically ill adults with acute respiratory failure.

**Methods:**

In this single-center, prospective, observational study, adults aged 18–75 years with acute respiratory failure requiring invasive mechanical ventilation and a PaO_2_/FiO_2_ ratio >150 mmHg were enrolled within 24 h of ICU admission. Airway occlusion pressure at 100 ms (P_0.1_) and occlusion pressure (P_occ_) were measured at baseline and at 12, 24, 36, and 48 h. Calculated respiratory muscle pressure (P_mus_) and calculated transpulmonary driving pressure (ΔP_L_) were calculated from P_occ._ Median values over the first 48 h represented exposure. The primary outcome was 28-day ventilator-free days (VFDs). Secondary outcomes included 28-day mortality, oxygenation changes, and correlations with Richmond Agitation-Sedation Scale scores. Multivariable Poisson and Cox regression analyses were performed.

**Results:**

A total of 206 patients were included. Patients within prespecified preferred ranges (P_0.1_ 1.5–3.5 cmH_2_O, calculated P_mus_ 5–10 cmH_2_2O, and calculated ΔP_L_ ≤20 cmH_2_O) had more 28-day VFDs than those with low or high values. In multivariable Poisson regression, low and high P_0.1_, low calculated P_mus_, and high calculated ΔP_L_ were independently associated with fewer VFDs. In multivariable Cox regression adjusted for age, immunocompromised status, peak airway pressure, and APACHE II score, calculated ΔP_L_ >20 cmH_2_O was independently associated with increased 28-day mortality (hazard ratio 6.57, 95% confidence interval 2.29–18.86; *P* < 0.001). Both low and high P_0.1_ were also independently associated with mortality (hazard ratios 3.75 and 4.81, respectively). Oxygenation improved in patients with preferred effort levels, whereas ΔP_L_ >20 cmH_2_O was associated with new-onset hypoxemia. Richmond Agitation-Sedation Scale scores correlated most strongly with calculated P_mus_ (r = 0.76), followed by P0.1 (r = 0.50) and ΔPL (r = 0.43).

**Conclusions:**

Early respiratory drive and inspiratory effort within preferred physiological ranges were associated with more VFDs and lower mortality. Calculated ΔP_L_ showed the strongest association with adverse outcomes, supporting bedside monitoring of drive and effort during assisted ventilation.

**Trial Registration:**

NCT06433076. Registered 29 May 2024, retrospectively registered.

## Introduction

Mechanical ventilation provides essential respiratory support; however, the degree of ventilator assistance, whether excessive or insufficient, may significantly impact patient outcomes.

Excessive ventilatory support or frequent sedation and neuromuscular blocking agents can impair diaphragm function, leading to muscle atrophy and prolonging mechanical ventilation [1]. Goligher et al. reported that changes in diaphragm thickness fraction, measured by ultrasound within the first week, correlate with prolonged ventilation, extended intensive care unit (ICU) stays, and complications [2]. A thickness fraction of 15–30% during the first three days was associated with the shortest ventilation duration, offering guidance for respiratory support management. Conversely, excessive patient inspiratory effort can contribute to additional lung injury through multiple mechanisms [3]. Elevated transpulmonary pressures and large tidal volumes increase global lung stress and strain [4]. Strong inspiratory drive and effort may also lead to patient-ventilator asynchronies, such as double triggering and breath stacking, which can cause inadvertent overdistension. Despite protective ventilation settings, localized injury may occur in dependent lung regions due to atelectasis and heterogeneous mechanical behavior. Vigorous diaphragmatic contractions during assisted or spontaneous breathing can generate large pleural pressure swings, leading to intrapulmonary gas redistribution known as Pendelluft. Pendelluft refers to the movement of gas between lung regions with differing alveolar pressures, resulting in no net change in tidal volume delivered by the ventilator. This phenomenon results in an uneven distribution of ventilation, with preferential inflation of dependent lung regions. The “occult” pendelluft can impose excessive regional stress and strain [5]. These changes also elevate transmural vascular pressure, increasing the risk of pulmonary edema, potentially contributing to patient self-inflicted lung injury (P-SILI) [4]. Additionally, experimental data suggest that excessive inspiratory load can lead to diaphragmatic inflammation and injury [6].

Although the electrical activity of the diaphragm and esophageal pressure are considered gold standards for evaluating respiratory drive and effort, their practical application is primarily confined to research settings. For clinical purposes, at the bedside, pressure generated within the airway 100 milliseconds after the onset of an inspiratory effort against an occluded airway (P_0.1_) is a dependable indicator of respiratory drive and helps detect abnormal inspiratory efforts; however, occlusion pressure (P_occ_) may be more effective in identifying cases of excessive respiratory effort.

Our study hypothesized that both insufficient and excessive respiratory drive and effort in patients with respiratory failure may be associated with critical clinical outcomes such as ventilator-free days (VFDs) and mortality. To investigate this hypothesis, we conducted a study exploring the relationship between bedside inspiratory drive and effort parameters and these key clinical outcomes.

## Methods

### Study design

A single-center, observational, prospective study was conducted between June 2022 and April 2024. The Research Ethics Committee approved the study protocol (COA. MURA2022/317). The research adhered to the ethical standards outlined in the Declaration of Helsinki and Good Clinical Practice guidelines. Informed consent was obtained from all participants or their legal representatives.

### Patients

We enrolled patients with acute respiratory failure requiring mechanical ventilation within 24 h of ICU admission at Ramathibodi Hospital, Mahidol University. Respiratory failure was classified according to the conventional clinical classification as type I (hypoxemic), type II (hypercapnic), type III (atelectatic/perioperative), or type IV (shock-related) [7,8]. The inclusion criteria were: (1) age between 18 and 75. This age range was prespecified in the study protocol and approved by the ethics committee to avoid inclusion of very old, potentially vulnerable patients. (2) a partial pressure of arterial oxygen (P)/fraction of inspired oxygen (F) ratio above 150. Patients with severe acute respiratory distress syndrome (ARDS) were excluded separately because they often require deep sedation, neuromuscular blockade, prone positioning, or extracorporeal membrane oxygenation, which could substantially affect the physiological assessment of respiratory drive and effort. The other exclusion criteria were no informed consent, history of prior hospitalization for 1 month, reintubation in the same admission, pregnancy, end-stage cancer, acute myocardium ischemia and/or cerebral ischemia 1 month before enrollment, active neuropsychiatric disease, comatose, status epilepticus, uncontrol thyroid disease, extubated before 48 h after enrollment, post thoracic and abdominal surgery or required intercostal tube drainage. Patients with acute myocardial ischemia, stroke/cerebral ischemia, active psychiatric disease, or uncontrolled thyroid disease were excluded because these conditions may independently influence respiratory drive and effort through non-respiratory mechanisms.

### Study protocol

Demographic data, type of respiratory failure, vital signs, Sequential Organ Failure Assessment (SOFA) score, Acute Physiology and Chronic Health Evaluation (APACHE) II score, comorbidities, intubation date, laboratory results, and gas exchange values were recorded for all participants. Patients were initially ventilated using assisted volume-controlled ventilation. During the measurement of inspiratory drive and effort parameters, the tidal volume was set at 6–8 ml/kg of predicted body weight (PBW), ensuring plateau pressure and airway driving pressure remained ≤30 cm H_2_O and ≤15 cm H_2_O, respectively. The Richmond Agitation-Sedation Scale (RASS) and inspiratory drive and effort parameters, including P_0.1_ and Pocc, were measured at 0, 12, 24, 36, and 48 h after enrollment, using assisted volume-controlled ventilation.

During the data collection period, assisted volume-controlled ventilation or assisted pressure-controlled ventilation was applied and set according to the attending physician's adjustment. Sedation and analgesia were managed according to the local protocol, based on the RASS and the Behavioral Pain Scale (BPS) [9]. However, the target sedation and pain levels were individualized by the attending physician rather than strictly mandated by the study protocol. Detailed data on sedative type, dose, and cumulative exposure were not systematically collected for the present analysis. The inspiratory effort parameters were not monitored or known to the attending physician.

### Primary and secondary outcomes

The primary outcome was to determine the association between inspiratory effort parameters during the first 48 h of mechanical ventilation and 28-day VFDs. Secondary outcomes included determining the association between inspiratory effort during the first 48 h and 28-day hospital mortality, gas exchange, and the correlation between inspiratory effort parameters and the RASS. We also assessed new-onset hypoxemia during the first 7 days after enrollment. New-onset hypoxemia was defined as a newly documented hypoxemic event within 7 days after enrollment, as assessed by the attending physicians, after exclusion of predefined alternative explanations such as progression of the primary pulmonary disease or underlying clinical condition, active airway obstruction, volume overload, and pulmonary embolism.

### Measurement

Inspiratory drive and effort parameters were serially assessed at five prespecified time points during the first 48 h after enrollment: at baseline (T0), within the first 24 h of ICU admission, and at 12 (T1), 24 (T2), 36 (T3), and 48 (T4) hours after the baseline assessment.

P_0.1_ was measured using the ventilators’ built-in function (Puritan Bennett™ 840 or Hamilton G5). Because the two ventilator platforms use different internal methods to estimate P_0.1_, the measurement approach was not fully identical across devices [10]. In particular, the Hamilton G5 displays breath-by-breath P_0.1_ derived from analysis of the airway pressure waveform, extrapolated to 100 ms, whereas the Puritan Bennett ventilator uses its built-in occlusion-based measurement function.

P_occ_ was obtained using the built-in negative inspiratory force function on the same ventilators. This function enabled an end-expiratory occlusion maneuver, allowing patients to generate negative airway pressure without assistance from the mechanical ventilator [11].

At each time point, five measurements of both P_0.1_ and P_occ_ were recorded, and the three closest values were averaged for further calculations. For each patient, the median of these averaged values across the five time points (T0, T1, T2, T3, and T4) was used to represent early exposure. This patient-level median was then used to assign patients to the predefined P_0.1_, calculated P_mus_, and calculated ΔP_L_ subgroups.

Patients who did not complete the 48 -h assessment period were excluded from the final subgroup analysis, and no imputation was performed for missing serial measurements.

Calculated respiratory muscle pressure (P_mus_) and calculated transpulmonary driving pressure(ΔP_L_) were calculated using formulas proposed by Bertoni et al. [12]: Calculated P_mus_ = - (3/4 × ΔP_occ_) and calculated ΔP_L_ = (Peak airway pressure - PEEP) - (2/3 × ΔP_occ_). P_0.1_ and calculated P_mus_ were designated as inspiratory drive and effort parameters in this study.

Thresholds for respiratory drive and inspiratory effort were selected *a priori* from published physiologic literature [12,13]. A P_0.1_ of 1.5–3.5 cmH_2_O and a calculated P_mus_ of 5–10 cmH_2_O were considered the preferred ranges; values below and above these ranges were classified as low and high respiratory drive/effort, respectively. P_occ_ was not categorized using a separate threshold, but was used to calculate calculated P_mus_ and calculated ΔP_L_ according to the method described by Bertoni et al. Because Bertoni et al. noted uncertainty regarding the optimal definition of excessive dynamic transpulmonary driving pressure and evaluated two possible thresholds (15 and 20 cmH_2_O), we prespecified calculated ΔP_L_ >20 cmH_2_O as the threshold for the high-ΔP_L_ group in order to use a more conservative cutoff for clearly excessive dynamic lung stress during assisted ventilation [12,13].

### Statistical analysis

The required sample size was calculated using data on ventilated patients in the ICU at Ramathibodi Hospital. Specifically, Zα/2Z_{\alpha/2}Zα/2​ was set at 1.96, corresponding to a 95% confidence level(CI) (α = 0.05), and ZβZ_\betaZβ​ was set at 0.84, corresponding to a power of 80% (β = 0.2). The population variance (σ2\sigma^2σ2) was 121.874 days [2]. The hypothesized clinically significant difference in VFDs was defined as 5 days. Based on these inputs, the calculated sample size required for the study was 154 subjects.

The data are presented as mean ± SD, median with (interquartile range), or percentages, as appropriate. Differences between two groups of continuous data were assessed using either a t-test or the Mann-Whitney U test, depending on the distribution of the data. Differences among three groups of continuous data were analyzed using ANOVA for normally distributed variables, while the Kruskal-Wallis test was used for non-normally distributed variables. Unadjusted mortality proportions were initially compared across subgroups using Pearson’s chi-square test. Additional pairwise comparisons were subsequently performed using the prespecified preferred subgroup as the reference category, and the results are presented as odds ratios (ORs) with 95% confidence intervals (CIs) and corresponding p-values.

As an exploratory supplementary analysis, we examined whether inspiratory drive and effort variables tended to vary in the same direction at the individual-patient level. Scatter plots were constructed using patient-level median values over the first 48 h for P_0.1_ versus P_occ_, calculated P_mus_, and calculated ΔP_L_, and the associations were assessed using Spearman’s rank correlation coefficient. Correlations between RASS, respiratory effort variables, and 28-day VFDs were also evaluated using Spearman’s correlation.

Multivariate Poisson regression was used to evaluate the effect of inspiratory effort on 28-day VFDs. A multivariate Cox regression analysis was performed for predictive factors associated with 28-day mortality. Multicollinearity among independent variables was assessed using the variance inflation factor (VIF) and tolerance. Collinearity diagnostics were examined for the final multivariable models, with particular attention to peak airway pressure, respiratory system compliance, and calculated ΔP_L_, because of their physiological relatedness. A VIF value less than 10 was considered no problematic collinearity.

As a sensitivity analysis, we fitted additional Poisson and Cox regression models using the a priori physiology-driven covariate set for both outcomes, including tidal volume, PEEP, driving pressure, respiratory system compliance, and peak airway pressure. Because P_0.1_, calculated P_mus_, and calculated ΔP_L_ are physiologically related, each exposure variable was entered into separate models. This sensitivity analysis was performed to assess the robustness of the findings to an alternative model specification based on physiological plausibility rather than outcome-specific univariable screening.

Because ventilator-free days and death are competing outcomes, we performed a competing-risk analysis using cause-specific Cox proportional hazards models. Successful liberation from invasive mechanical ventilation within 28 days was modeled as the event of interest, and death before liberation was treated as a competing event. Patients alive but not liberated at day 28 were censored at day 28. A separate cause-specific model was also fitted for death before liberation. Time to liberation was derived from 28-day ventilator-free days, and death time was derived from the recorded duration to mortality. Each exposure was modeled separately and adjusted for age, APACHE II score, and immunocompromised status. Kaplan–Meier survival analysis was performed to compare survival distributions between subgroups based on respiratory effort.

Statistical significance was defined as a p-value < 0.05. All statistical analyses were conducted using STATA version 17 and SPSS version 30.

## Results

**Fig. 1:**
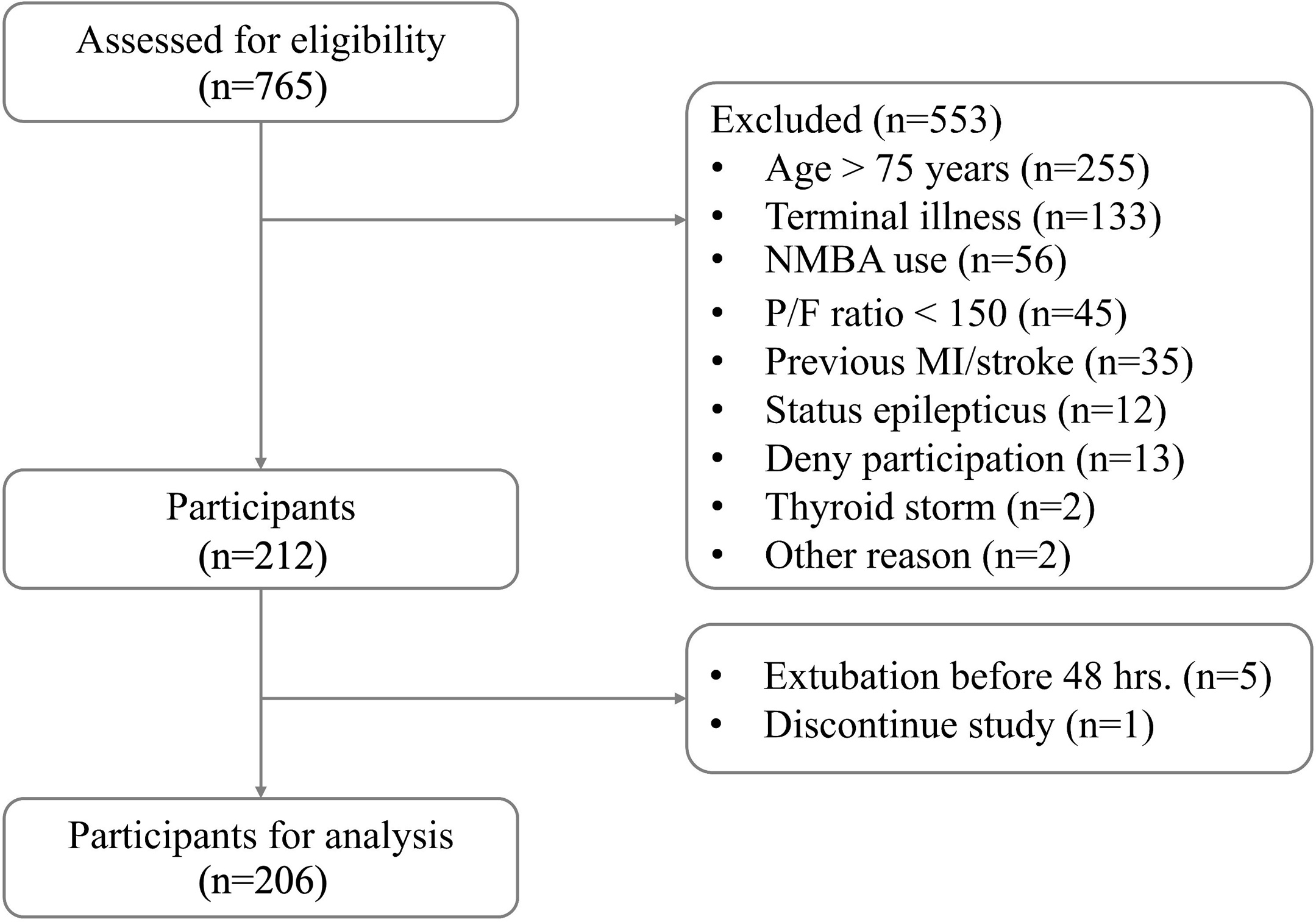
Shows the flow diagram of participants included in the study.

A total of 765 mechanically ventilated patients were enrolled; after excluding 559, 206 were included in the analysis (Fig. 1). The average age of the patients was 58 ± 15 years, with 58.3% of the patients being male. The average initial APACHE-II score was 16 ± 6. Type I respiratory failure was identified in 51.9% of cases. The median P/F ratio was 301 (245−393). Immunocompromised individuals accounted for 30.6% of cases. Overall, 34 patients (16.5%) died within 28 days. (Table 1). Table 2 displays patient characteristics and comparisons within subgroups based on inspiratory drive and effort parameters.

**Table 1:** Baseline characteristics.

Variables	All (N = 206)
Age, mean (SD), years	58 ± 15
Gender, n (%)	
Female	86 (41.7)
BMI, mean (SD), kg/m^2^	24.1 ± 7.3
Smoker, n (%)	93 (45.1)
Co-morbid disease, n (%)	
Airway diseases	60(29.1)
ILD	10(4.9)
Heart diseases	78(37.8)
Neurological diseases	62(30.0)
Kidney diseases	88(42.7)
Immunocompromised	63(30.6)
Malnutrition status, n (%)	103 (50.0)
COVID-19, n (%)	16 (7.8)
Multiorgan damage, n (%)	5 (2.4)
Obesity, n (%)	41 (19.9)
APACHE-II, mean (SD)	16 ± 6
SOFA score, mean (SD)	6 ± 3
Tidal volume, mean (SD), ml/kg	7.62 ± 1.52
MV, mean (SD), L/min	7.57 ± 1.51
PEEP, mean (SD), cm H_2_O	7.6 ± 2.7
Driving pressure, mean (SD), cm H_2_O	10.1 ± 4.1
Cause of respiratory failure, n (%)	
Type I	107(51.9)
Type II	43(20.9)
Type III	2(0.9)
Type IV	54(26.2)
Crs, median (IQR), ml/cm H_2_O	44 (33−60)
Rrs, median (IQR), cmH_2_O-sec/L	5 (3−8)
P_0.1_, median (IQR), cmH_2_O	1.5(0.8−2.2)
P_occ_, median (IQR), cmH_2_O	7.8(5.8−11.5)
Calculated P_mus_, median (IQR), cmH_2_O	5.8(4.4−8.6)
Calculated ΔP_L_, median (IQR), cmH_2_O	18.3(14.7, 22.7)
P/F ratio, median (IQR)	301(245−393)
pH, mean (SD),	7.37 ± 0.11
RASS, median (IQR)	−1(-2−0)
Hb, mean (SD), g/dL	10.05 ± 2.64
Lactate, median (IQR), mmol/L	2.5(1.4−4.7)
28-day mortality, n (%)	34(16.5)

Abbreviations: SD, standard deviation; IQR, interquartile range; BMI, Body Mass Index; ILD, Interstitial Lung Disease; APACHE II, Acute Physiology and Chronic Health Evaluation II; SOFA, Sequential Organ Failure Assessment; MV, Minute Ventilation; PEEP, Positive End-Expiratory Pressure; CrsRespiratory System Compliance; Rrs, Respiratory System Resistance; P0.1, Airway Occlusion Pressure at 100 milliseconds; Pmus, Inspiratory Muscle Pressure; ΔP_L_, Transpulmonary Driving Pressure; P/F ratio, Partial Pressure of Arterial Oxygen to Fraction of Inspired Oxygen; RASS, The Richmond Agitation-Sedation Scale; Hb, Hemoglobin.

**Table 2:** Compares baseline characteristics among subgroups categorized by P_0.1_, calculated P_mus_, or calculated ΔP_L._

	P_0.1_				Calculated P_mus_				Calculated ΔP_L_		
Variables	<1.5 (N = 107)	1.5−3.5 (N = 82)	>3.5 (N = 17)	*P*	<5 (N = 80)	5−10 (N = 93)	>10 (N = 33)	*P*	≤20 (N = 141)	>20 (N = 65)	*P*
Age, mean (SD), years	59.5 ± 13.8	55.5 ± 16.9	59.4 ± 16.6	0.182	59.7 ± 13.4	56.3 ± 16.4	58.0 ± 16.8	0.352	58.5 ± 14.9	56.6 ± 16.4	0.420
BMI, mean (SD), kg/m^2^	23.19 ± 7.09	25.57 ± 7.82	23.16 ± 5.24	0.072	23.51 ± 7.96	24.59 ± 7.31	24.36 ± 5.65	0.616	24.4 ± 8.08	23.56 ± 5.33	0.445
APACHE-II, mean (SD)	17 ± 6	14 ± 7	16 ± 6	0.037	16 ± 6	15 ± 6	16 ± 7	0.427	15 ± 6	17 ± 6	0.053
SOFA score, mean (SD)	6 ± 2	5 ± 3	7 ± 5	0.044	7 ± 3	6 ± 3	6 ± 4	0.087	6 ± 3	7 ± 3	0.007
Tidal volume, mean (SD), ml/kg	7.60 ± 1.48	7.64 ± 1.61	7.73 ± 1.46	0.942	7.72 ± 1.36	7.45 ± 1.51	7.87 ± 1.89	0.305	7.72 ± 1.55	7.42 ± 1.47	0.190
MV, mean (SD), L/min	7.69 ± 1.23	7.31 ± 1.69	8.09 ± 1.96	0.080	7.72 ± 1.29	7.35 ± 1.43	7.84 ± 2.08	0.158	7.4 ± 1.3	7.94 ± 1.83	0.016
PEEP, mean (SD), cm H_2_O	8.3 ± 2.9	6.8 ± 2.3	6.9 ± 1.7	<0.001	8.5 ± 2.9	6.9 ± 2.3	7.3 ± 2.2	<0.001	7.5 ± 2.7	7.8 ± 2.6	0.436
Driving pressure, mean (SD), cm H_2_O	11.1 ± 4.1	8.9 ± 3.9	9.4 ± 3.6	0.001	11.4 ± 4.0	9.2 ± 4.1	9.5 ± 3.7	0.001	8.9 ± 3.5	12.7 ± 4.2	<0.001
Crs, median (IQR), ml/cm H_2_O	40(32−50)	45(34−72)	47(40−60)	0.018	40(30−50)	37(34−64)	48(34−60)	0.012	41(38−62)	34(26−45)	<0.001
Rrs, median (IQR), cmH_2_O-sec/L	6(4−9)	4(3−6)	4.8(3−7)	0.001	7(5−10.05)	4(3–6)	4(3–7)	<0.001	4.8(3−7.2)	6(4−9)	0.089
P/F ratio at enrollment, median (IQR)	297(250–370)	317(243–407)	338(293−389)	0.459	310(245–393)	295(240−382)	346(273−367)	0.404	300 (245−382)	255 (213–355)	0.992
Lactate, median (IQR), mmol/L	2.4(1.2–4.5)	2.5(1.4–3.7)	5.5(3.0–10.0)	0.004	2.5(1.3–4.8)	2.3(1.4−3.)	3.3(2.0−6.2)	0.106	2.4(1.4−4.0)	3(1.4−7.1)	0.082

Abbreviations: P0.1: Airway Occlusion Pressure at 100 milliseconds; Pmus: Inspiratory Muscle Pressure; ΔP_L_: Transpulmonary Driving Pressure; SD: standard deviation; IQR: interquartile range; BMI: Body Mass Index; ILD: Interstitial Lung Disease; APACHE II: Acute Physiology and Chronic Health Evaluation II; SOFA: Sequential Organ Failure Assessment; MV: Minute Ventilation; PEEP: Positive End-Expiratory Pressure; Crs: Respiratory System Compliance; Rrs: Respiratory System Resistance; P/F ratio: Partial Pressure of Arterial Oxygen to Fraction of Inspired Oxygen; Hb: Hemoglobin.

### The inspiratory effort parameters during the first 48 h of mechanical ventilation and the 28-day ventilator-free days

**Fig. 2:**
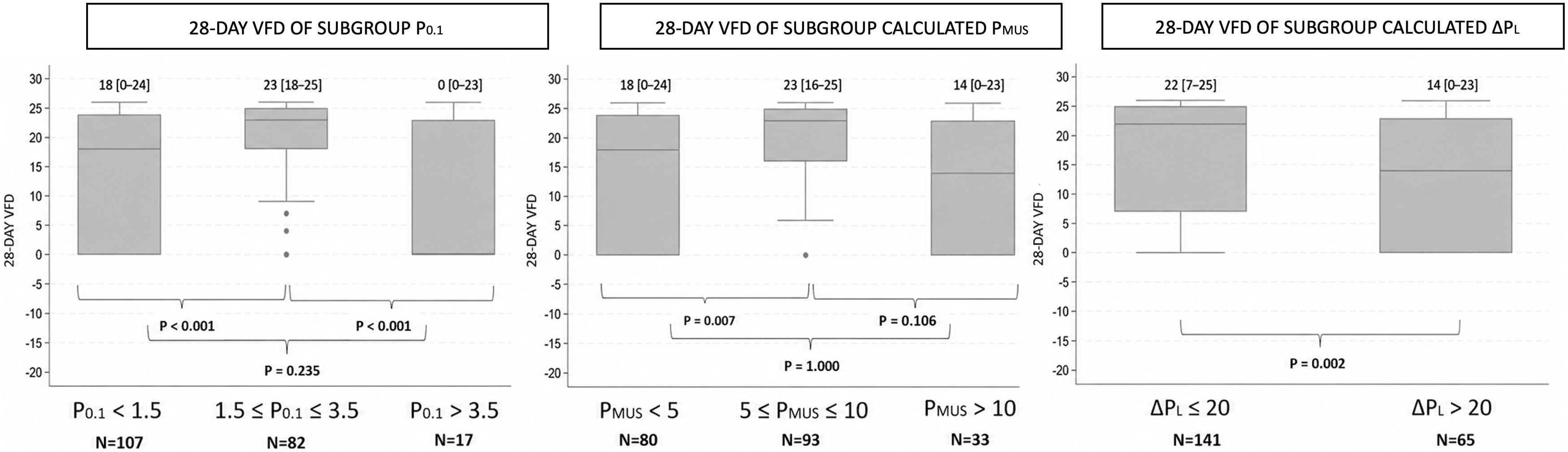
28-Day ventilator-free days (VFDs) according to early respiratory drive and inspiratory effort subgroups. Box-and-whisker plots show 28-day VFDs stratified by (A) airway occlusion pressure at 100 ms (P0.1: <1.5 cmH_2_O [n = 107], 1.5–3.5 cmH_2_O [n = 82], >3.5 cmH_2_O [n = 17]); (B) calculated respiratory muscle pressure (calculated Pmus: <5 cmH_2_O [n = 80], 5–10 cmH_2_O [n = 93], >10 cmH_2_O [n = 33]); and (C) calculated transpulmonary driving pressure (calculated ΔPL: ≤20 cmH_2_O [n = 141] vs >20 cmH_2_O [n = 65]). Values above boxes represent median [interquartile range]. The box denotes the interquartile range, the horizontal line indicates the median, whiskers represent dispersion, and points indicate outliers. Brackets and P values indicate between-group comparisons as displayed in the figure.

For P_0.1_, a significantly more 28-day VFDs was observed in the subgroup with the P_0.1_ between 1.5–3.5 cm H_2_O compared to those with above 3.5 cm H_2_O and lower than 1.5 cm H_2_O (23 [18–25] vs. 0 [0–23] days, *P* < 0.001, and 23 [18–25] vs. 18 [0–24] days, *P* < 0.001, respectively). No statistical difference was found between the groups with the P_0.1,_ those above 3.5 cm H_2_O, and those lower than 1.5 cm H_2_O (Fig. 2).

Patients with calculated P_mus_ within 5−10 cm H_2_O had a significantly higher 28-day VFDs compared to those with <5 cmH_2_O (23 [16–25] vs. 18 [0–24] days, *P* = 0.007). However, no significant difference was observed between those with P_mus_ within 5−10 cm H_2_O with >10 cmH_2_O (23 [16–25] vs. 14 [0–23] days, *P* = 0.106) and calculated P_mus_ >10 cmH_2_O with <5 cmH_2_O (14 [0–23] vs. 18 [0–24] days, *P* = 1.00).

For calculated ΔP_L_, the subgroup with the calculated ΔP_L_ ≤20 cmH_2_O value had a significantly higher 28-day VFDs compared to the other subgroup (22 [7–25] vs. 14 [0–23] days, *P* = 0.002).

Multivariate Poisson regression, utilizing all significant univariate variables, included gender (male), age, body mass index, interstitial lung disease, immunocompromised status, malnutrition, respiratory system compliance, peak airway pressure, and APACHE II score, confirmed P_0.1_, calculated P_mus_, and calculated ΔP_L_ as independent predictors of 28-day VFDs. Specifically, low and high inspiratory effort based on P_0.1_ were associated with lower VFDs (incidence rate ratio [IRR] 0.82, 95% CI 0.75−0.90, *P* < 0.001, and IRR 0.54, 95% CI 0.45−0.65, *P* < 0.001, respectively). Similarly, high calculated ΔP_L_ also demonstrated lower VFDs (IRR, 0.77; 95% CI, 0.69−0.86; *P* < 0.001). Although low effort based on calculated P_mus_ was associated with lower VFDs (IRR 0.89, 95% CI 0.81–0.98, *P* < 0.021), high effort based on calculated P_mus_ was not (IRR 1.03, 95% CI 0.91–1.17, *P* = 0.651), as shown in Table 3.

**Table 3:** Univariate and multivariate analysis of primary outcomes (28-day ventilator-free day).

	Univariate analysis			Multivariate analysis in all Models		
Variables	IRR, 95%CI	Z	*P*	IRR, 95%CI	Z	*P*
Model 1						
Male	0.84 (0.79, 0.90)	−4.83	<0.001	0.90 (0.83, 0.97)	−2.61	0.009
Age, years	0.99 (0.99, 0.99)	−8.06	<0.001	0.99 (0.99, 1.00)	−6.61	<0.001
BMI, kg/m^2^	1.02 (1.01, 1.02)	7.69	<0.001	1.00 (1.00, 1.00)	1.12	0.261
ILD	0.53 (0.42, 0.66)	−5.77	<0.001	0.66(0.52, 0.82)	−3.65	<0.001
Immunocompromise status	0.81 (0.75, 0.87)	−5.32	<0.001	0.81 (0.75, 0.89)	−4.74	<0.001
Malnutrition	0.68 (0.63, 0.73)	−10.78	<0.001	0.78(0.72, 0.84)	−6.28	<0.001
Crs, ml/cm H_2_O	1.00 (1.00, 1.00)	6.94	<0.001	1.00 (1, 1.003)	2.36	0.018
Peak pressure, cm H_2_O †	0.98 (0.97, 0.99)	−7.74	<0.001	1.00 (0.99, 1.01)	−0.13	0.893
Enrolled APACHE II, score †	0.83 (0.74−0.89)	−4.78	<0.001	1.00 (0.99−1.00)	−0.61	0.54
Model 2						
1.5≤P_0.1_≤3.5 cm H_2_O (Preference)	Reference			Reference		
P_0.1_<1.5 cm H_2_O	0.68 (0.63, 0.73)	−10.65	<0.001	0.82 (0.75, 0.90)	−4.36	<0.001
P_0.1_>3.5 cm H_2_O	0.44 (0.37, 0.52)	−9.48	<0.001	0.54 (0.45, 0.65)	−6.51	<0.001
Model 3						
5≤Pmus≤10 cm H_2_O (Preference)	Reference			Reference		
P_mus_<5 cm H_2_O	0.73 (0.68, 0.79)	−8.02	<0.001	0.89 (0.81, 0.98)	−2.3	0.021
P_mus_>10 cm H_2_O †	0.75 (0.68, 0.84)	−5.35	<0.001	1.03 (0.91, 1.17)	0.45	0.651
Model 4						
ΔP_L_≤ 20 cm H_2_O (Preference)	Reference			Reference		
ΔP_L_>20 cm H_2_O	0.72 (0.66, 0.78)	−8.11	<0.001	0.77 (0.69, 0.86)	−4.61	<0.001

IRR < 1 indicates that the factor is associated with a reduced incidence rate of ventilator-free days, corresponding to a decrease of (1 - IRR) × 100%, which reflects worse outcomes when compared to the reference factor.

A Variance Inflation Factor (VIF) of less than 5 across all variables typically indicates acceptable levels of multicollinearity.

Abbreviations: IRR: incidence rate ratio; P0.1: Airway Occlusion Pressure at 100 milliseconds; Pmus: Inspiratory Muscle Pressure; ΔP_L_: Transpulmonary Driving Pressure; BMI: Body Mass Index; ILD: Interstitial Lung Disease; Rrs: Respiratory System Resistance; APACHE II: Acute Physiology and Chronic Health Evaluation II.

† Although all significant in univariate Poisson regression, this parameter was not independently associated with 28-day ventilator-free days in the all-input variables multivariate model.

In sensitivity analyses using the physiology-driven covariate set—tidal volume, PEEP, driving pressure, respiratory system compliance, and peak airway pressure—in the Poisson models, the main findings were generally unchanged. Low and high _P0.1_, both low and high calculated P_mus,_ and calculated ΔP_L_ >20 cmH_2_O remained associated with fewer 28-day VFDs. (Supplementary Table S1)

### The inspiratory effort parameters during the first 48 h of mechanical ventilation and 28-day mortality

**Fig. 3:**
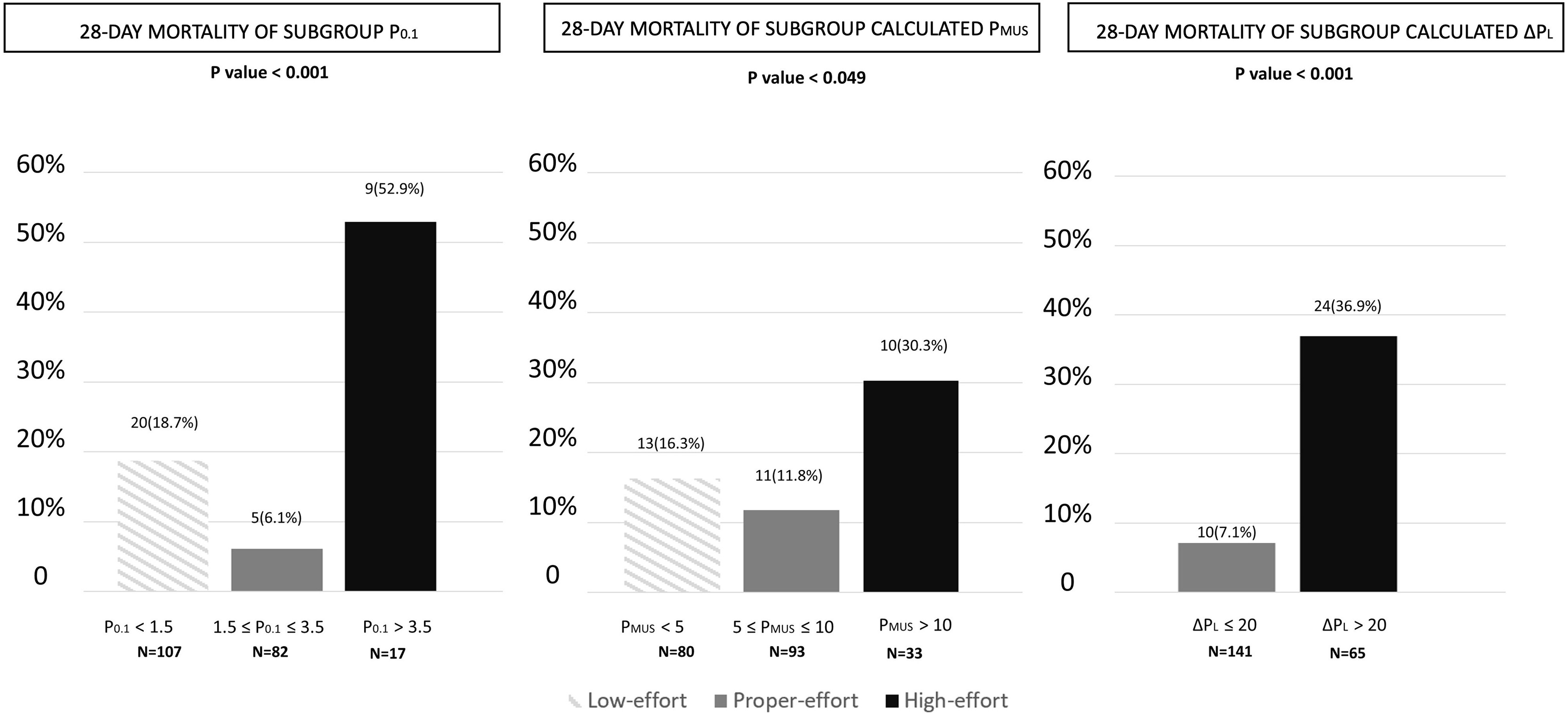
28-Day mortality according to early respiratory drive and inspiratory effort subgroups. Values above bars indicate the number of patients in each subgroup and the corresponding mortality proportion. P values above each panel represent overall between-group comparisons. Shading indicates low-, proper-, and high-effort categories as shown in the legend.

Twenty-eight-day mortality differed significantly across P_0.1_ categories, being lowest in patients with P_0.1_ 1.5–3.5 cmH_2_O and highest in those with P_0.1_ >3.5 cmH_2_O: 6.1% and 52.9%, respectively (χ^2^ = 23.19, *P* < 0.001). Compared with the reference P_0.1_ 1.5–3.5 group, P_0.1_ <1.5 was associated with higher mortality [Odds ratio(OR) 3.54, 95% CI 1.27–9.89; *P* = 0.016], whereas P_0.1_ >3.5 was associated with a markedly higher mortality risk (OR 17.32, 95% CI 4.66–64.43; *P* < 0.001) (Fig. 3 and Supplementary Table S2).

Mortality also differed across P_mus_ categories: 11.8% in P_mus_ 5–10 cmH_2_O, 16.3% in P_mus_ <5 cmH_2_O, and 30.3% in P_mus_ >10 cmH_2_O (χ^2^ = 6.04, *P* = 0.049). Compared with P_mus_ 5–10 cmH_2_O, high Pmus >10 cmH_2_O was associated with significantly higher mortality (OR 3.24, 95% CI 1.22–8.58; *P* = 0.027), whereas low Pmus <5 cmH_2_O was not significantly different (OR 1.45, 95% CI 0.61–3.44; *P* = 0.509). (Fig. 3 and Supplementary Table S2)

Patients with calculated ΔP_L_ >20 cmH_2_O had substantially higher 28-day mortality than those with calculated ΔP_L_ ≤20 cmH_2_O: 36.9% versus 7.1% (χ^2^ = 28.73, *P* < 0.001). The odds of 28-day mortality were approximately 7.7-fold higher in the calculated ΔP_L_ >20 group (OR 7.67, 95% CI 3.39–17.36; *P* < 0.001) (Fig. 3 and Supplementary Table S2).

Multivariate Cox regression analysis, which included age, immunocompromised status, Peak pressure, and APACHE II score, revealed that the calculated ΔP_L_ was identified as the independent predictor of 28-day mortality (Hazard Ratio [HR] 6.57, 95% CI (2.29–18.86), *P* < 0.001). Likewise, both low P_0.1_ and high P_0.1_ were significantly associated with 28-day mortality (HR 3.75, 95% CI 1.03–13.62, *P* = 0.045; HR 4.81, 95% CI 1.50–15.42, *P* = 0.008, respectively) (Table 4).

**Table 4:** Univariate and multivariate Cox regression analysis of 28-day mortality.

Variables	Univariate analysis			Multivariate analysis (all-input model)		
	HR (95% CI)	Z	*P*	HR (95% CI)	Z	*P*
Model 1						
Age, years	1.03 (1.00, 1.06)	2.07	0.038	1.05 (1.02, 1.08)	2.82	0.005
Enrolled APACHE II, score †	1.06 (1.01, 1.12)	2.40	0.016	1.05 (0.99, 1.11)	1.59	0.111
Immunocompromise	1.88 (0.96, 3.68)	1.83	0.067	3.15 (1.49, 6.68)	3.00	0.003
Peak pressure, cm H_2_O †	1.07 (1.02, 1.13)	2.71	0.007	1.013 (0.93, 1.10)	0.30	0.761
Model 2						
1.5≤P_0.1_≤3.5 cm H_2_O (Preference)	Reference			Reference		
P_0.1_<1.5 cm H_2_O	2.86 (1.07–7.61)	2.09	0.036	3.75 (1.03, 13.62)	2.01	0.045
P_0.1_>3.5 cm H_2_O	11.16(3.73–33.37)	4.29	<0.001	4.81(1.50, 15.42)	2.65	0.008
Model 3						
5≤P_mus_≤10 cm H_2_O (Preference)	Reference			Reference		
P_mus_<5 cm H_2_O	1.26 (0.56, 2.81)	0.56	0.577	1.331 (0.46, 3.90)	0.52	0.602
P_mus_>10 cm H_2_O †	2.82 (1.20, 6.66)	2.37	0.018	1.78 (0.55, 5.71)	0.96	0.335
Model 4						
ΔP_L_≤20 cm H_2_O (Preference)	Reference			Reference		
ΔP_L_>20 cm H_2_O	6.33 (3.02–13.29)	4.84	<0.001	6.57 (2.29, 18.86)	3.50	<0.001

Abbreviations: P0.1: Airway Occlusion Pressure at 100 milliseconds; Pmus: Inspiratory Muscle Pressure; ΔP_L_: Transpulmonary Driving Pressure; APACHE II: Acute Physiology and Chronic Health Evaluation II; HR: hazard ratio; CI: confidence interval.

† Although significant in Univariate analysis, this parameter was not independently associated with 28-day mortality in the multivariate model.

In sensitivity analyses using the physiology-driven covariates, high P_0.1_, calculated P_mus_ >10 cmH_2_O, and calculated ΔP_L_ >20 cmH_2_O associated with increased 28-day mortality, whereas the associations of low P_0.1_ and calculated P_mus_ with mortality were not statistically significant (Supplementary Table S3).

In the final multivariable models, collinearity diagnostics did not indicate problematic multicollinearity for either 28-day VFDs or 28-day mortality (Supplementary Table S4)

**Fig. 4:**
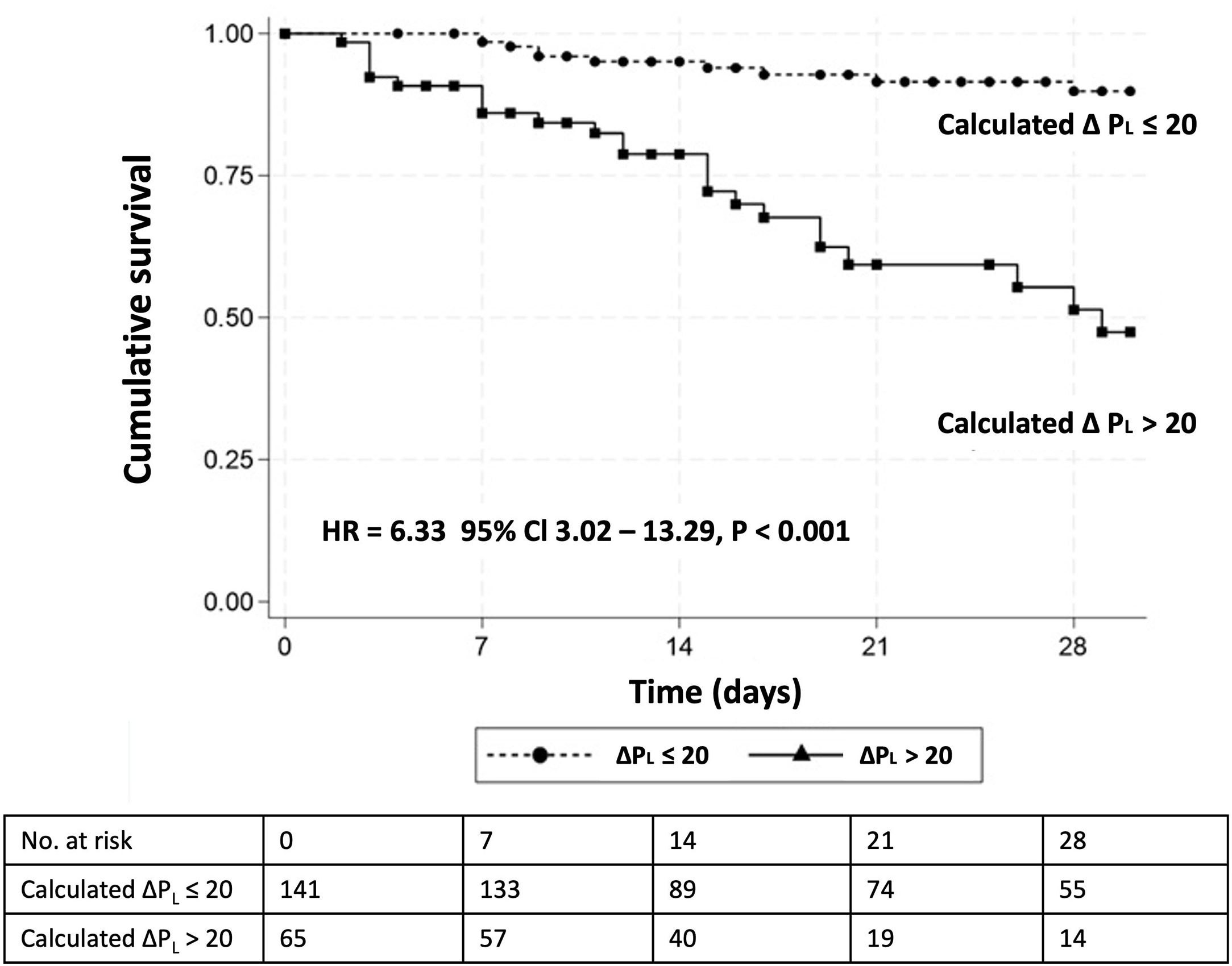
Presents the 28-day survival analysis based on the calculated ΔP_L_ groups using the Kaplan–Meier method. The cumulative survival rate from the initiation of mechanical ventilation to day 28 was significantly lower in patients with a calculated ΔP_L_ greater than 20 cmH_2_O (*P* < 0.001).

**Fig. 5:**
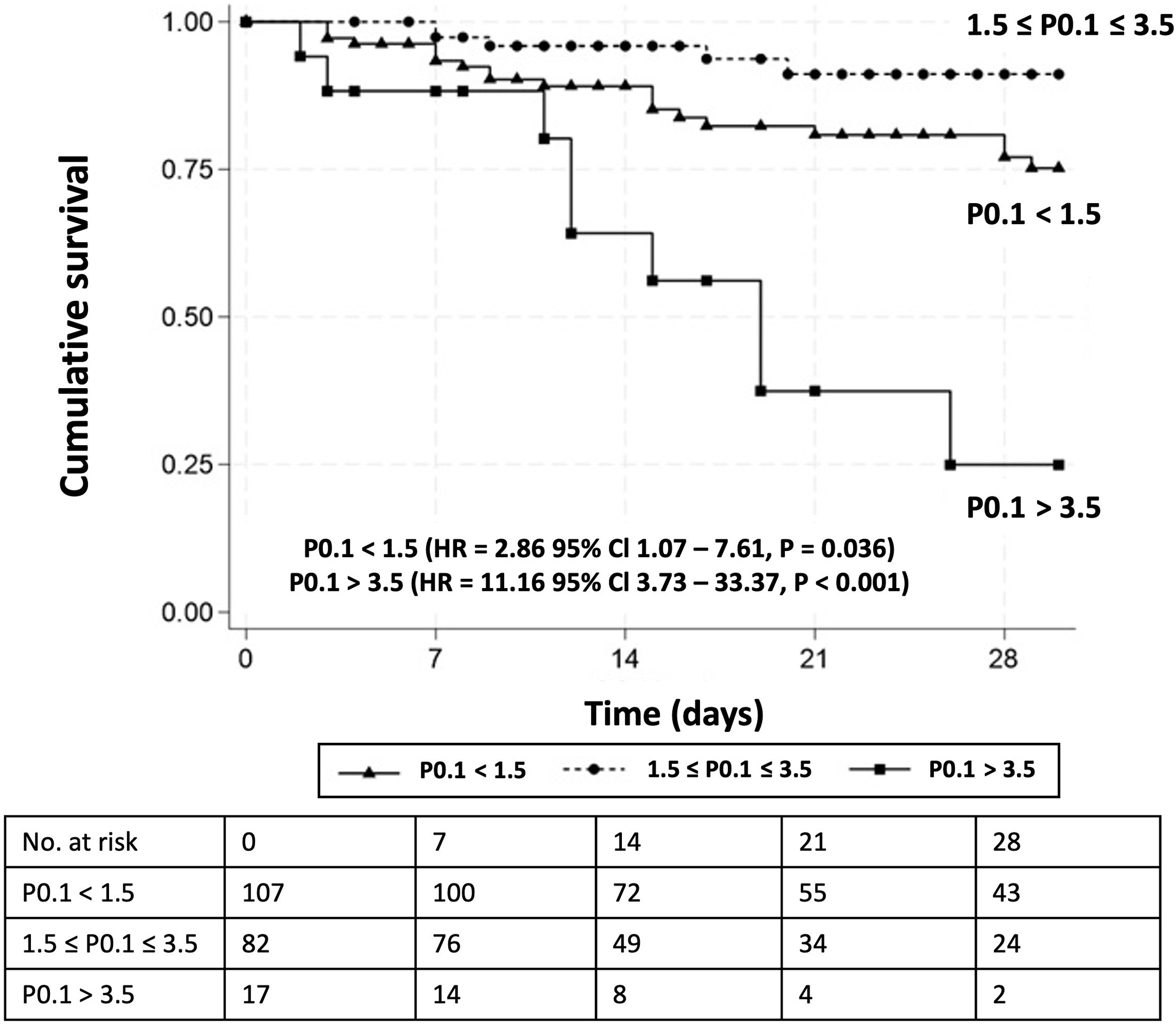
Presents the 28-day survival analysis based on the P0.1 groups using the Kaplan–Meier method. The cumulative survival rate from the initiation of mechanical ventilation to day 28 was significantly lower in patients with a calculated P0.1 < 1.5 (*P* = 0.036) and > 3.5 cmH_2_O (*P* < 0.001).

The Kaplan–Meier curve shows significantly lower 28-day cumulative survival in patients with calculated ΔP_L_ >20 cmH_2_O compared with those with ΔP_L_ ≤20 cmH_2_O (HR = 6.33, 95% Cl 3.02–13.29; *P* < 0.001) (Fig. 4). Fig. 5 demonstrate graded differences in 28-day cumulative survival across P_0.1_ categories, with the highest survival in the reference range (1.5–3.5 cmH_2_O) and significantly lower survival in patients with low P_0.1_(<1.5 cmH_2_O; HR 2.86, 95% CI 1.07–7.61; *P* = 0.036) and especially high P_0.1_ (>3.5 cmH_2_O; HR 11.16, 95% CI 3.73–33.37; *P* < 0.001).

### Competing-risk analysis of liberation from invasive mechanical ventilation and death

Among 206 patients, 149 (72.3%) were successfully liberated from invasive mechanical ventilation by day 28, 34 (16.5%) died before liberation, and 23 (11.2%) were alive but not liberated at day 28. In adjusted cause-specific Cox models, calculated ΔP_L_ >20 cmH_2_O was associated with both a lower hazard of successful liberation (adjusted HR 0.69, 95% CI 0.49–0.98; *P* = 0.037) and a higher hazard of death before liberation (adjusted HR 6.39, 95% CI 2.93–13.91; *P* < 0.001). P_0.1_ >3.5 cmH_2_O was similarly associated with delayed liberation and a higher death-specific hazard. These findings were directionally consistent with the primary analysis of 28-day ventilator-free days (Supplementary Tables S5 and S6).

### Exploratory analysis of ICU and hospital length of stay according to inspiratory drive and effort subgroups

As an exploratory supplementary analysis, ICU length of stay and hospital length of stay were compared across the predefined P_0.1_, calculated P_mus_, and calculated ΔP_L_ subgroups using pairwise nonparametric comparisons (Mann–Whitney U test). The results showed no significant differences in ICU length of stay across P_0.1_, calculated P_mus_, or calculated ΔP_L_ categories. Hospital length of stay also did not differ significantly across P_0.1_ or calculated P_mus_ subgroups, with a significant difference observed only for calculated ΔP_L_ subgroups (*P* = 0.043). (Supplementary Figure S1)

### The effect of inspiratory effort parameters on gas exchange

We observed significant improvements in the PF ratio at 48 h after enrollment in several subgroups: P_0.1_ 1.5–3.5 cmH_2_O (334 ± 146–385 ± 183; *P* = 0.023), calculated P_mus_ 5–10 cmH_2_O (322 ± 136–386 ± 181; *P* < 0.001), and calculated ΔP_L_ ≤20 cmH_2_O (334 ± 121–369 ± 142; *P* = 0.047) Supplementary Figure S2.

In total, 25% of mechanically ventilated patients developed new-onset hypoxemia within 1 week after excluding worsening disease, active airway obstruction, volume overload, and pulmonary embolism. New-onset hypoxemia was independently associated with high respiratory drive and effort (P_0.1_>3.5 cmH_2_O; OR 4.71, 95% CI 1.53–14.48; *P* < 0.001) and calculated ΔP_L_ >20 cmH_2_O (OR 4.00, 95% CI 1.97–8.10; *P* < 0.001).

### Correlation between the inspiratory effort parameters and RASS score

Over 48 h, the median RASS scores correlated significantly with inspiratory effort indices, showing a strong correlation with the median calculated P_mus_ (r = 0.76, *P* < 0.001) and moderate correlations with P_0.1_ (r = 0.50, *P* < 0.001) and calculated ΔP_L_ (r = 0.43, *P* = 0.008) (Supplementary Figure S3).

### Correlation between P_0.1_ and other inspiratory drive and effort parameters

In an exploratory patient-level correlation analysis using median 48 -h values, P_0.1_ showed a strong positive correlation with P_occ_ and calculated P_mus_ (Spearman r = 0.78 and 0.79, respectively, for both, *P* < 0.001), and a weaker but still significant positive correlation with calculated ΔP_L_ (r = 0.30, *P* < 0.001) (Supplementary Figure S4 and Table S7).

## Discussion

In this cohort of mechanically ventilated adults aged 18–75 years with acute respiratory failure and a P/F ratio >150 mmHg, the main findings of our study were as follows: The main findings of our study were as follows: (1) Patients with P_0.1_ values within the preferred range of 1.5–3.5 cmH_2_O had significantly longer 28-day VFDs compared to those with lower or higher P_0.1_ values. Patients with calculated Pmus within 5−10 cm H_2_O had a significantly higher 28-day VFDs compared to those with <5 cmH_2_O, furthermore, calculated ΔP_L_ ≤20 cmH_2_O experienced longer VFDs than patients in other subgroups; (2) Multivariate Poisson regression analysis confirmed that low and high inspiratory effort based on P_0.1_, low effort based on calculated P_mus,_ and high calculated ΔP_L_ were independent predictors of reduced VFDs; nevertheless, high calculated Pmus did not significantly affect VFDs. (3) Regarding mortality, Multivariate Cox regression analysis, which included age, immunocompromised status, peak pressure, and APACHE II score, calculated a ΔP_L_ >20 cmH_2_O, low P_0.1,_ and high P_0.1_ remained a significant independent predictor of increased 28-day mortality. (4) Oxygenation significantly improved at 48 h in patients with optimal inspiratory effort values across all three parameters. Notably, a calculated ΔP_L_ >20 cmH_2_O and P_0.1_>3.5 cmH_2_O were associated with new-onset hypoxemia during the first week after excluding other causes. Finally, (5) A strong correlation was also observed between the RASS score and calculated P_mus_, while the correlation with P_0.1_ and calculated ΔP_L_ was moderate.

Clinical approaches to mechanical ventilation often emphasize strategies to prevent ventilator-induced lung injury, primarily by minimizing lung stress and strain during fully controlled modes. However, similar injury mechanisms—including elevated transpulmonary pressures and excessive tidal volumes—can also arise during assisted ventilation but may not receive equivalent attention [14]. Intense spontaneous inspiratory efforts can contribute to P-SILI [5,10] and diaphragmatic damage (myotrauma), both of which are linked to prolonged mechanical ventilation [2] and increased rates of morbidity and mortality [15]. Achieving safe spontaneous breathing presents a considerable challenge, as it involves striking a balance: mitigating transpulmonary pressure and tidal volume to reduce the risk of P-SILI, while maintaining sufficient respiratory effort to prevent diaphragm disuse and subsequent atrophy [16].

A study by Bertoni et al. showed that spontaneously breathing patients under mechanical ventilation often exceed established safety thresholds for inspiratory effort and dynamic lung stress, regardless of the ventilatory mode or sedation level. Few patients demonstrated the ideal protective combination of parameters for lung and diaphragm health (P_mus_ ≤10 cm H_2_O and ΔP_L_ ≤20 cm H_2_O). Moreover, airway pressures displayed on the ventilator frequently underestimated the true extent of lung stress, underscoring the limitations of relying solely on ventilator-derived data to assess mechanical loads during spontaneous breathing [12].

Several studies reported that the optimal P_0.1_ value reflects the inspiratory effort that balances assisted ventilation and sedation. Telias et al. reported that P_0.1_ and pressure-time product (PTP) [13] were strongly correlated. The P_0.1_ over 3.5 is associated with potentially excessive effort of PTP greater than or equal to 200 cm H_2_O/min [17]. The over-assisted ventilation, defined by work of breathing less than 0.3 J/L or PTP less than 50 cm H_2_O/min, was associated with P_0.1_. In COVID-19 patients during pressure support ventilation, higher P_0.1_ values are associated with prolonged duration of invasive ventilation and ICU mortality, with the cut-off values of P_0.1_ for predicting VFDs <14 days and ICU mortality were 2.0 and 3.5, respectively [18].

A recent longitudinal cohort study by Dianti et al. reported associations between respiratory drive/effort and clinical outcomes during mechanical ventilation; however, these relationships differed according to oxygenation severity [19]. In patients with a P/F ratio >150 mmHg, higher P_0.1_ and Pocc were associated with accelerated ICU discharge. On the other hand, the non-linear pattern was more evident in those with more severe hypoxemia (P/F ratio ≤150). Both low and high P_0.1_, and both low and high Pocc, were associated with a lower rate of ICU discharge.

In contrast, in our cohort restricted to patients with P/F ratio >150 mmHg, intermediate respiratory drive, as reflected by P_0.1_ values of 1.5–3.5 cmH_2_O, was associated with the most favorable outcomes, whereas both low and high P_0.1_ were associated with worse 28-day VFDs and higher mortality. This difference may relate to differences in study population, severity of illness, exposure definition, outcomes of interest, and analytical approach. Our study summarized respiratory drive and effort using the median of five intermittent measurements obtained during the first 48 h after enrollment, whereas clinical outcomes were assessed at 28 days. Therefore, the observed associations should be interpreted as relationships between early physiologic exposure and later outcomes, rather than as direct evidence of a sustained mechanistic effect throughout the full course of mechanical ventilation. This differs from Dianti et al., who analyzed daily time-varying measurements over the first 10 days of ventilation, and this difference in exposure definition may partly explain the discrepancy between the two studies [19].

Furthermore, the magnitude of the associations observed in our study should be interpreted cautiously. The high-P_0.1_ subgroup comprised only 17 patients, yet showed the largest effect estimates. Therefore, the observed associations in this subgroup may reflect limited precision and potential model instability at a small sample size, and the corresponding effect sizes may overestimate the true magnitude of association.

Our cohort had relatively preserved baseline oxygenation, with a median baseline P/F ratio of 301 (245–393). Therefore, these findings should be interpreted in the context of a relatively mild hypoxemic population and may not be directly generalizable to patients with more severe hypoxemia. Although our cohort had relatively preserved oxygenation and near-normal respiratory mechanics, abnormal respiratory drive and effort were still frequently observed. This suggests that drive and effort during assisted ventilation are influenced not only by hypoxemia and respiratory mechanics but also by systemic illness severity, metabolic demand, acid-base status, sedation/arousal state, pain, delirium, and other non-respiratory factors. In our cohort, the high-P_0.1_ subgroup showed a trend toward higher lactate and SOFA scores than the reference group, and RASS correlated with inspiratory effort parameters, particularly calculated P_mus_. However, delirium was not systematically recorded, and agitation was not independently assessed beyond RASS; therefore, their contributions could not be directly determined.

A low P_0.1_ may indicate insufficient respiratory drive, potentially complicating weaning from mechanical ventilation [20]. Although we used P_0.1_ <1.5 cmH_2_O to define low respiratory drive, alternative lower cutoffs have been reported. Telias et al. found that P_0.1_ at reference ≤1.0 cmH_2_O identified low inspiratory effort (PTPmus ≤50 cmH_2_O·s·min^−1^) [17], highlighting that the optimal threshold for low effort remains uncertain and may depend on the physiologic definition and measurement method used.

The ideal P_mus_ during assisted ventilation remains unclear; however, recent evidence indicates that maintaining P_mus_ at levels resembling those of healthy individuals at rest (5–10 cmH_2_O) could be beneficial by reducing the risk of diaphragm atrophy [2,13]. More recent outcome-based data from Dianti et al. suggest that both insufficient and excessive respiratory drive/effort are associated with worse outcomes, supporting the concept that intermediate levels of effort may be most favorable [19]. We confirmed that the subgroup that calculated P_mus_ between 5–10 cmH_2_O had shorter VFDs than the others. Although the difference in 28-day VFDs between the calculated Pmus 5–10 cmH_2_O and >10 cmH_2_O groups did not reach statistical significance, the numerical difference was substantial and may reflect limited power in the smaller high-Pmus subgroup rather than the absence of a clinically relevant effect.

While elevated ΔP_L_ has been associated with an increased risk of ventilator-induced lung injury in ARDS [21], safe upper limits for ΔP_L_ in spontaneously breathing patients without ARDS have not been clearly established. In our study, lower 28-day mortality was observed in patients with P_0.1_ and calculated P_mus_ within the prespecified preferred ranges, as well as in those with calculated ΔP_L_ ≤20 cmH_2_O. Calculated ΔP_L_ >20 cmH_2_O and P_0.1_ >3.5 cmH_2_O were also associated with new-onset hypoxemia during the first week after enrollment, suggesting that excessive respiratory drive, inspiratory effort, and lung stress may be clinically relevant during assisted ventilation. However, this association should be interpreted cautiously because causality cannot be inferred from this observational study, and deterioration in oxygenation may also reflect progression of the underlying disease or other unmeasured factors. Moreover, although statistically significant improvements in PF ratio at 48 h were observed in some subgroups within the preferred range, these changes were modest and occurred in patients with relatively preserved baseline PF ratios; thus, their clinical relevance remains uncertain.

Importantly, an additional competing-risk analysis treating death before liberation as a competing event yielded results that were directionally consistent with the primary analysis. In particular, calculated ΔP_L_ >20 cmH_2_O remained associated with both delayed liberation from invasive mechanical ventilation and a higher hazard of death before liberation, supporting the robustness of the main findings.

An additional point requiring cautious interpretation is that the mortality associations were not entirely stable across model specifications. In the sensitivity analysis using the physiology-driven covariate set, the associations of low P_0.1_ and calculated P_mus_ with 28-day mortality were no longer statistically significant, whereas high P_0.1_, calculated P_mus_ >10 cmH_2_O, and calculated ΔP_L_ >20 cmH_2_O remained associated with mortality. This suggests that some of the mortality findings, particularly for low P_0.1_ and calculated P_mus_, may be model-dependent and should be interpreted cautiously.

Our study demonstrated strong positive correlations between RASS scores and median calculated P_mus_ over 48 h, suggesting a more consistent relationship between clinical sedation assessment and objective measures of respiratory drive and effort than previously reported. Furthermore, we found moderate correlations between RASS and P_0.1_ and between RASS and calculated ΔP_L_. In contrast to the earlier study that demonstrated frequent discordance between sedation depth and respiratory effort [17], our findings indicate that RASS scores aligned with physiological measurements of inspiratory effort in this patient population.

## Limitation

This study has several limitations. First, as a single-center, prospective observational study, the findings may have limited generalizability and should be interpreted as associations rather than causation. Second, subgroup analyses were underpowered—especially in the excessive effort group, potentially reflecting the prevalence of heavy sedation—and the key lung-stress metric (calculated ΔP_L_) was calculated from ΔP_occ_ using a specific formula. While correlated with transpulmonary pressure swing, esophageal manometry remains the gold standard for the most accurate assessment of pleural pressure. Third, we enrolled only patients aged 18–75 years, as prespecified in the study protocol and approved by the ethics committee. Therefore, our findings may not be generalizable to older patients, particularly those aged 75 years or older, who may have different respiratory drive, ventilatory responses, comorbidity burden, and clinical outcomes.

Fourth, our cohort had relatively preserved oxygenation at baseline, with a median P/F ratio of 301 (245–393), and we enrolled only patients with a P/F ratio >150 while excluding those with severe ARDS. This exclusion strategy was intended to reduce major non-pulmonary determinants of respiratory drive and effort and to allow serial physiologic assessment during assisted ventilation in a more comparable cohort; however, it also resulted in a selected lower-risk population with relatively preserved oxygenation and lower mortality. Accordingly, our findings should be interpreted cautiously and may not be generalizable to patients with more severe hypoxemia, including severe ARDS, or to those requiring deep sedation, neuromuscular blockade, prone positioning, or ECMO. We excluded patients whose conditions may independently drive respiratory effort through non-pulmonary pathways — such as stroke, acute myocardial ischemia, or thyrotoxicosis — to reduce the risk that observed poor outcomes were driven by the severity of the primary illness rather than by respiratory effort itself. However, this approach has an important limitation: from a physiological standpoint, elevated inspiratory effort injures the lung regardless of what triggers it. By excluding these patients, we may have narrowed our findings to a more homogeneous but less representative population, and our results should not be extrapolated to unselected ICU patients in whom respiratory drive may be elevated for multiple coexisting reasons.

Fifth, inspiratory drive and effort were summarized using the median of five intermittent measurements during the first 48 h rather than modeled as time-varying or cumulative exposures. Therefore, temporal changes and cumulative burden over time may not have been fully captured, and the mechanistic interpretation of associations between early exposure and 28-day outcomes should be made cautiously.

Sixth, although sedation and analgesia were managed according to a local protocol, targets were not fully standardized because target RASS and pain levels were individualized by the attending physicians. Thus, variation in clinician-directed sedation and analgesia may have influenced inspiratory drive and effort, as well as clinical outcomes. In addition, detailed data on sedative type, dose, and cumulative exposure were unavailable for adjustment. Consequently, residual confounding may have remained, as patients with lower inspiratory effort may have received higher sedative doses either because they were more severely ill or because deep sedation itself may adversely affect outcomes. Seventh, P_0.1_ was measured using two different ventilator platforms with different internal measurement algorithms. Device-specific differences in P_0.1_ acquisition may have introduced measurement heterogeneity. In particular, continuous/extrapolated P_0.1_ measurements, such as those used by Hamilton-G5, have been reported to be less accurate than occlusion-based measurements in a bench study [10] and may exhibit device-dependent bias. Therefore, absolute P_0.1_ values should be interpreted with caution. Finally, ventilator mode and settings, as well as sedative dosing, were adjusted at the discretion of attending physicians. Although inspiratory effort parameters were not visible to clinicians, unmeasured practice variation may still have influenced effort and clinical outcomes.

## Clinical applications

Clinically, these parameters should be interpreted together: P_0.1_ as a marker of respiratory drive, calculated P_mus_ as a marker of inspiratory muscle effort, and calculated ΔP_L_ as a marker of dynamic lung stress. Because discordance may occur within the same patient, no single parameter should be used in isolation. Instead, when feasible, the practical goal should be to maintain all parameters within their prespecified preferred ranges. Such discordance may indicate that respiratory drive, inspiratory muscle effort, and lung stress are not fully aligned in a given patient. In this context, no single value should outweigh the overall clinical assessment; rather, integrating these measures may help identify whether the predominant concern is excessive respiratory drive, excessive inspiratory effort, or excessive lung stress.

## Conclusion

In mechanically ventilated patients, maintaining early respiratory drive and inspiratory effort within preferred physiological ranges (P_0.1_ 1.5–3.5 cmH_2_O, P_mus_ 5–10 cmH_2_O, and calculated ΔP_L_ ≤20 cmH_2_O) was associated with more ventilator-free days and lower mortality. Among the evaluated bedside measures, calculated ΔP_L_ showed the strongest association with adverse outcomes, underscoring the potential importance of monitoring lung stress during assisted ventilation. These findings support integrating bedside assessment of respiratory drive and effort to optimize ventilatory support and sedation, with the goal of preventing both diaphragm disuse and lung injury.

## Authors’ contributions

Phruet Soipetkasem: Conceptualization, Data curation, Formal analysis, Investigation, Methodology, Project administration, Resources, Validation, Visualization, Writing – original draft, Writing – review & editing.

Touchapong Taksinwarajarn: Conceptualization, Data curation, Formal analysis, Investigation, Methodology, Project administration, Resources, Validation, Visualization, Writing – original draft, Writing – review & editing.

Detajin Junhasavasdikul: Methodology, supervision, Writing – original draft, Writing – review & editing.

Yuda Sutherasan: Conceptualization, Data curation, Formal analysis, Investigation, Methodology, Project administration, Resources, Supervision, Validation, Visualization, Writing – original draft, Writing – review & editing.

Pongdhep Theerawit: Conceptualization, Data curation, Formal analysis, Investigation, Methodology, Project administration, Resources, Supervision, Validation, Visualization, Writing – original draft, Writing – review & editing.

All authors have read and approved the final manuscript.

## Consent for publication

Not applicable. No individual personal data (e.g., images or videos) is included in this publication.

## Ethics approval and consent to participate

The study was approved by the Research Ethics Committee of Ramathibodi Hospital, Faculty of Medicine, Mahidol University (COA. MURA2022/317). Written informed consent was obtained from all participants or their legal representatives, in accordance with the Declaration of Helsinki and Good Clinical Practice guidelines.

## Declaration of Generative AI and AI-assisted technologies in the writing process

During the preparation of this work, the authors used ChatGPT and Grammarly to refine the language and ensure grammatical correctness. After using this service, the authors reviewed and edited the content as needed and take full responsibility for the final publication.

## Funding

This research received no specific grant from funding agencies in the public, commercial, or not-for-profit sectors.

## Availability of data and materials

The datasets generated and/or analyzed during the current study are available from the corresponding author on reasonable request.

## Declaration of competing interest

The authors declare that we have no competing interests.
