# Supplementary material for: Impact of Respiratory Effort Parameters on Clinical Outcomes in Respiratory Failure Patients (Effort-I): A Prospective Observational Study
Source: Ann Intensive Care. 2026 Jun 20;16:100103. doi: 10.1016/j.aicoj.2026.100103 (PMC13321231; doi:10.1016/j.aicoj.2026.100103)
Supplement: Supplementary file 1 [file mmc1.docx]

**Table of contents**

**Supplementary Figures**
- Supplementary Figure S1. ICU and hospital length of stay across predefined P_0.1_, calculated Pmus, and calculated ΔP_L_ subgroups.
- Supplementary Figure S2. Trajectory of oxygenation (PaO₂/FiO₂ ratio) over the first 48 hours according to early respiratory drive and inspiratory effort subgroups.
- Supplementary Figure S3. Correlation between sedation level and respiratory drive/effort parameters during the first 48 hours of mechanical ventilation.
- Supplementary Figure S4. Scatter plots showing the relationship of median P_0.1_ with median P_occ_, median calculated P_mus_, and median calculated ΔP_L_ across individual patients.

**Supplementary Table**

-Supplementary Table S1. Sensitivity analysis using physiology-driven Poisson regression models for 28-day ventilator-free days.

-Supplementary Table S2*.* Pairwise odds ratios for 28-day mortality across predefined P_0.1_, calculated P_mus_, and calculated ΔP_L_ subgroups

-Supplementary Table S3. Sensitivity analysis using physiology-driven Cox regression models for 28-day mortality

-Supplementary Table S4. Collinearity diagnostics for final multivariable models and physiology-driven sensitivity models.

-Supplementary Table S5. Cause-specific hazard of successful liberation from invasive mechanical ventilation by day 28

-Supplementary Table S6. Cause-specific hazard of death before liberation by day 28
-Supplementary Table S7. Spearman correlations between median P_0.1_ and other inspiratory drive/effort variables.

**Supplementary Figure S1**

**ICU(A) and hospital(B) length of stay across predefined P_0.1_, calculated P_mus_, and calculated ΔP_L_ subgroups.**

A
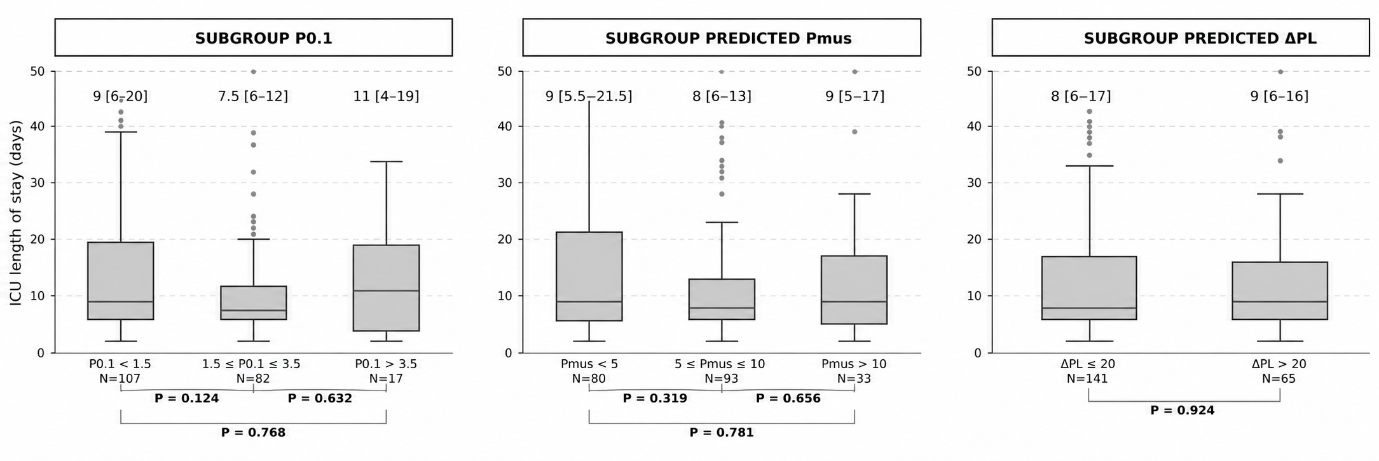


B


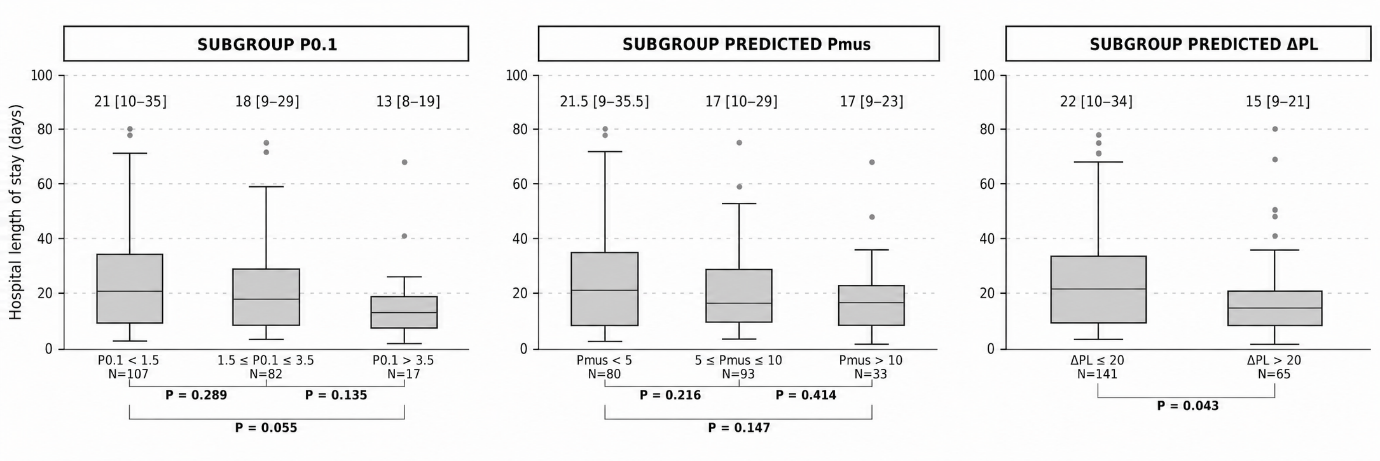


Box plots show ICU and hospital length of stay by subgroup. Data are presented as median [Q1–Q3]. Group comparisons were performed using nonparametric tests.

**Supplementary Figure S2.** **Trajectory of oxygenation (PaO₂/FiO₂ ratio) over the first 48 hours according to early respiratory drive and inspiratory effort subgroups.**
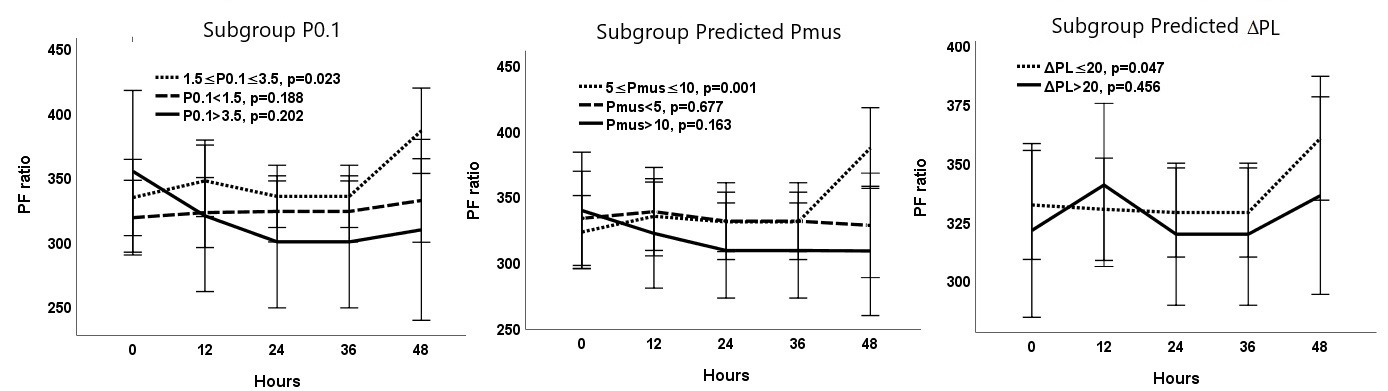


Line plots show PaO₂/FiO₂ (PF) ratio measured at 0, 12, 24, 36, and 48 hours after enrollment, left figure stratified by P_0.1_ subgroups (<1.5, 1.5–3.5, and >3.5 cmH₂O), middle figure: calculated P_mus_ subgroups (<5, 5–10, and >10 cmH₂O), and right figure: calculated ΔP_L_ subgroups (≤20 vs >20 cmH₂O). Symbols/line types correspond to the subgroup categories as indicated in each panel. Data points are shown with error bars to reflect variability at each time point. P values in each panel indicate the significance of the change in PF ratio over time within each subgroup.

**Supplementary Figure S3. Correlation between sedation level and respiratory drive/effort parameters during the first 48 hours of mechanical ventilation**


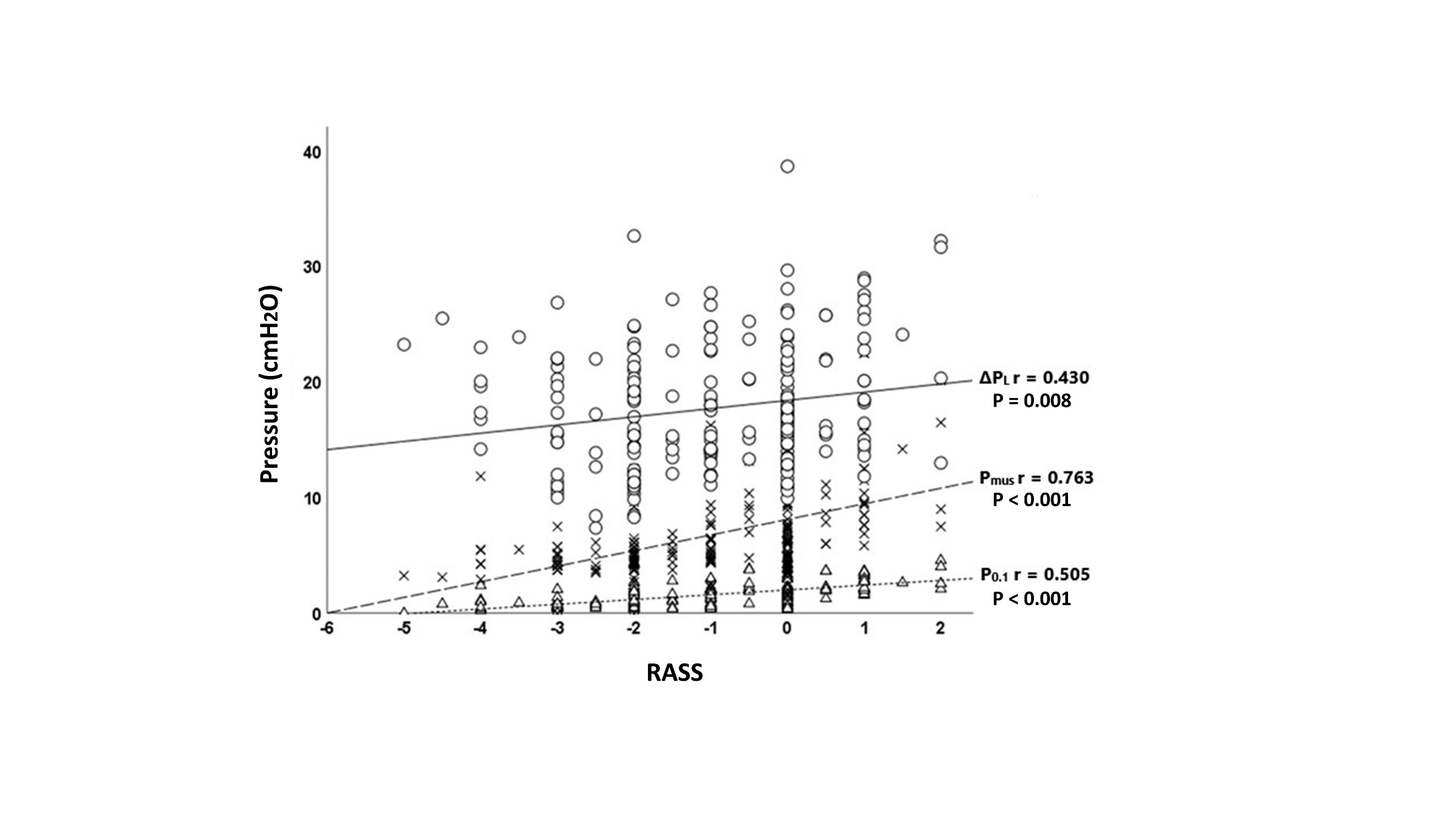


Scatter plots depict the relationships between the Richmond Agitation–Sedation Scale (RASS) and bedside measures of respiratory drive and effort, including airway occlusion pressure at 100 ms (P0.1), calculated respiratory muscle pressure (calculated Pmus), and calculated transpulmonary driving pressure (calculated ΔPL). Each point represents an observation, and fitted linear regression lines are shown for each parameter. The strength of association is reported as the correlation coefficient (r) with corresponding P values, demonstrating the strongest correlation for calculated Pmus (r = 0.763; P < 0.001), followed by P0.1 (r = 0.505; P < 0.001) and calculated ΔPL (r = 0.430; P = 0.008).

**Supplementary Figure S4.** Scatter plots showing the relationship of median P_0.1_ with median P_occ_ (4A), median calculated P_mus_ (4B), and median calculated ΔP_L (_4C) across individual patients.

A


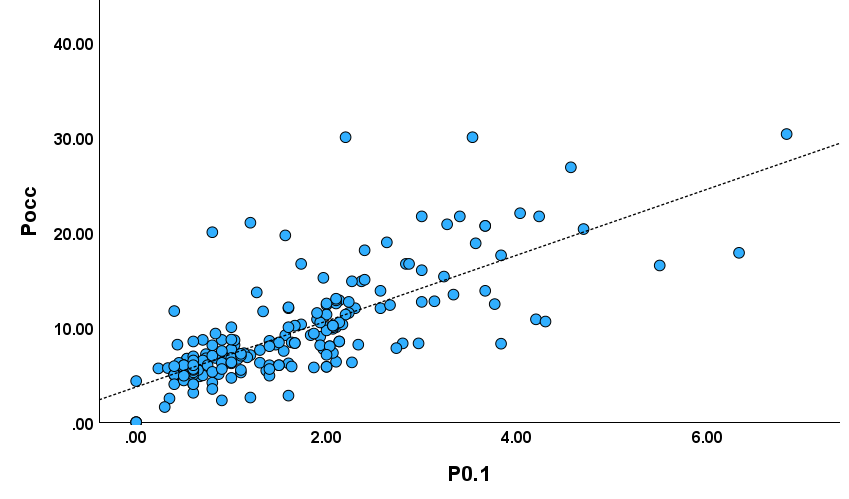


B


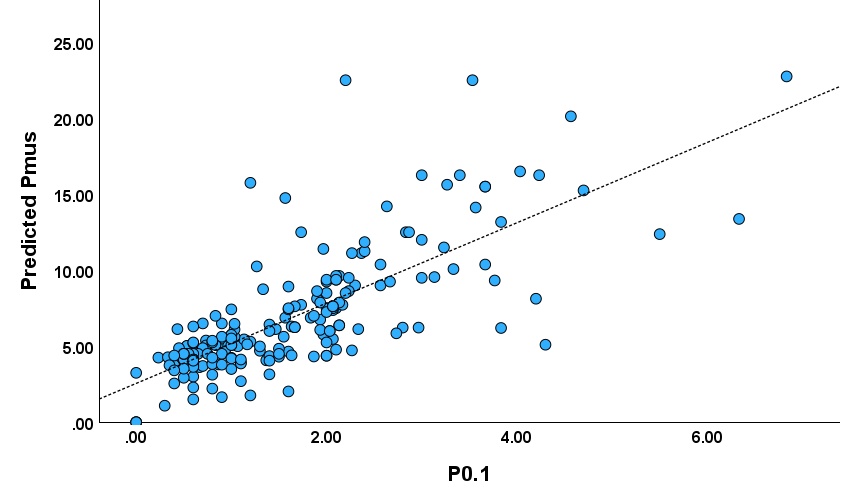


C


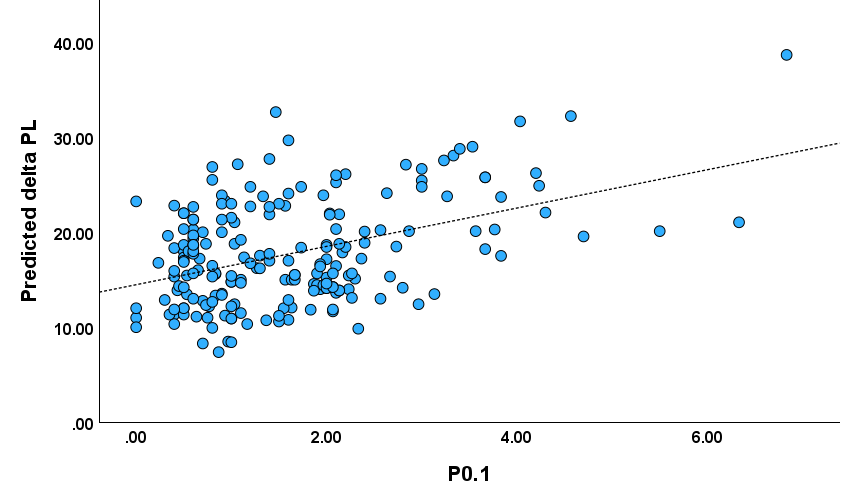


Supplementary Table S1. Sensitivity analysis using physiology-driven Poisson regression models for 28-day ventilator-free days

**A priori covariates included in each model:** tidal volume, PEEP, driving pressure, respiratory system compliance, and peak airway pressure.

Panel A. P_0.1_ model

| **Variable** | **Unadjusted IRR (95% CI)** | ***P*** | **Adjusted IRR (95% CI)** | ***P*** |
| --- | --- | --- | --- | --- |
| Tidal volume, mL/kg PBW | 1.05 (1.03–1.08) | <0.001 | 1.06 (1.04–1.09) | <0.001 |
| PEEP, cmH₂O | 0.97 (0.95–0.98) | <0.001 | 0.99 (0.96–1.01) | 0.136 |
| Driving pressure, cmH₂O | 0.97 (0.96–0.98) | <0.001 | 1.01 (0.99–1.04) | 0.301 |
| Respiratory system compliance, mL/cmH₂O | 1.00 (1.00–1.00) | <0.001 | 1.00 (1.00–1.01) | <0.001 |
| Peak airway pressure, cmH₂O | 0.98 (0.97–0.99) | <0.001 | 0.99 (0.98–1.00) | 0.166 |
| **P_0.1_ subgroup** |  |  |  |  |
| 1.5–3.5 cmH₂O | Reference | — | Reference | — |
| <1.5 cmH₂O | 0.68 (0.63–0.73) | <0.001 | 0.74 (0.68–0.80) | <0.001 |
| >3.5 cmH₂O | 0.44 (0.37–0.52) | <0.001 | 0.45 (0.38–0.53) | <0.001 |

Panel B. Calculated P_mus_ model

| **Variable** | **Unadjusted IRR (95% CI)** | ***P*** | **Adjusted IRR (95% CI)** | ***P*** |
| --- | --- | --- | --- | --- |
| Tidal volume, mL/kg PBW | 1.05 (1.03–1.08) | <0.001 | 1.07 (1.05–1.10) | <0.001 |
| PEEP, cmH₂O | 0.97 (0.95–0.98) | <0.001 | 0.98 (0.96–1.00) | 0.063 |
| Driving pressure, cmH₂O | 0.97 (0.96–0.98) | <0.001 | 1.01 (0.98–1.03) | 0.633 |
| Respiratory system compliance, mL/cmH₂O | 1.00 (1.00–1.00) | <0.001 | 1.00 (1.00–1.01) | <0.001 |
| Peak airway pressure, cmH₂O | 0.98 (0.97–0.99) | <0.001 | 0.99(0.98–1.01) | 0.259 |
| **Calculated P_mus_ subgroup** |  |  |  |  |
| 5–10 cmH₂O | Reference | — | Reference | — |
| <5 cmH₂O | 0.73 (0.68–0.79) | <0.001 | 0.80 (0.73–0.87) | <0.001 |
| >10 cmH₂O | 0.75 (0.68–0.84) | <0.001 | 0.75 (0.67–0.83) | <0.001 |

Panel C. Calculated ΔP_L_ model

| **Variable** | **Unadjusted IRR (95% CI)** | ***P*** | **Adjusted IRR (95% CI)** | ***P*** |
| --- | --- | --- | --- | --- |
| Tidal volume, mL/kg PBW | 1.05 (1.03–1.08) | <0.001 | 1.06 (1.03–1.08) | <0.001 |
| PEEP, cmH₂O | 0.97 (0.95–0.98) | <0.001 | 0.98 (0.97–1.00) | 0.120 |
| Driving pressure, cmH₂O | 0.97 (0.96–0.98) | <0.001 | 1.04 (1.02–1.06) | 0.002 |
| Respiratory system compliance, mL/cmH₂O | 1.00 (1.00–1.00) | <0.001 | 1.00 (1.00–1.01) | <0.001 |
| Peak airway pressure, cmH₂O | 0.98 (0.97–0.99) | <0.001 | 0.98(0.97–0.99) | 0.002 |
| **Calculated ΔP_L_ subgroup** |  |  |  |  |
| ≤20 cmH₂O | Reference | — | Reference | — |
| >20 cmH₂O | 0.72(0.66–0.78) | <0.001 | 0.76 (0.69–0.83) | <0.001 |

**Abbreviations:** IRR, incidence rate ratio; PBW, predicted body weight; PEEP, positive end-expiratory pressure; Driving pressure; P_mus_, calculated respiratory muscle pressure; ΔP_L_, calculated transpulmonary driving pressure.

**Supplementary Table S2**

Pairwise odds ratios for 28-day mortality across predefined P_0.1_, calculated P_mus_, and calculated ΔP_L_ subgroups

# **A .Overall statistical comparisons**

| **Variable** | **Comparison** | **Test** | **χ² (df)** | ***P*** |
| --- | --- | --- | --- | --- |
| P_0.1__3gr | Across P_0.1_ categories | Pearson’s chi-square | 23.19 (2) | <0.001 |
| Calculated P_mus__3gr | Across P_mus_ categories | Pearson’s chi-square | 6.04 (2) | 0.049 |
| Calculated ΔP_L_ _2gr | ΔP_L_>20 vs P_L_ ≤20 | Pearson’s chi-square | 28.73 (1) | <0.001 |

B. Odds ratios for 28-day mortality

| **Variable** | **Comparison** | **OR** | **95% CI** | ***P*** |
| --- | --- | --- | --- | --- |
| P_0.1__3gr | P_0.1_ <1.5 vs P_0.1_ 1.5–3.5 | 3.54 | 1.27–9.89 | 0.016 |
| P_0.1__3gr | P_0.1_ >3.5 vs P_0.1_ 1.5–3.5 | 17.32 | 4.66–64.43 | <0.001 |
| Calculated P_mus__3gr | P_mus_ <5 vs P_mus_ 5–10 | 1.45 | 0.61–3.44 | 0.509 |
| Calculated P_mus__3gr | P_mus_ >10 vs P_mus_ 5–10 | 3.24 | 1.22–8.58 | 0.027 |
| Calculated ΔP_L_ _2gr | ΔP_L_  20 vs ΔP_L_ ≤20 | 7.67 | 3.39–17.36 | <0.001 |

**Abbreviations:** OR, odds ratio;CI, confidence interval; Pmus, calculated respiratory muscle pressure; ΔPL, calculated transpulmonary driving pressure.

Supplementary Table S3. Sensitivity analysis using physiology-driven Cox regression models for 28-day mortality

**A priori covariates included in each model:** tidal volume, PEEP, driving pressure, respiratory system compliance, and peak airway pressure.

**Panel A. P_0.1_ model**

| **Variable** | **Unadjusted HR (95% CI)** | ***P*** | **Adjusted HR (95% CI)** | ***P*** |
| --- | --- | --- | --- | --- |
| Tidal volume, mL | 1.00 (0.99–1.01) | 0.956 | 1.00 (0.99–1.01) | 0.721 |
| PEEP, cmH₂O | 1.05 (0.94–1.18) | 0.377 | 1.00 (0.84–1.19) | 0.993 |
| Driving pressure, cmH₂O | 1.13 (1.04–1.23) | 0.003 | 1.07 (0.87–1.31) | 0.529 |
| Respiratory system compliance, mL/cmH₂O | 0.98 (0.96–1.00) | 0.026 | 1.00 (0.97–1.03) | 0.881 |
| Peak airway pressure, cmH₂O | 1.07 (1.02–1.13) | 0.006 | 1.04 (0.92–1.17) | 0.582 |
| **P_0.1_ subgroup** |  |  |  |  |
| 1.5–3.5 cmH₂O | Reference | — | Reference | — |
| <1.5 cmH₂O | 2.86 (1.07–7.61) | 0.036 | 2.037 (0.729–5.692) | 0.175 |
| >3.5 cmH₂O | 11.16(3.73–33.37) | <0.001 | 10.20 (3.40–30.63) | <0.001 |

**Panel B. Calculated P_mus_ model**

| **Variable** | **Unadjusted HR (95% CI)** | ***P*** | **Adjusted HR (95% CI)** | ***P*** |
| --- | --- | --- | --- | --- |
| Tidal volume, mL | 1.00 (0.99–1.01) | 0.956 | 1.00 (0.99–1.01) | 0.969 |
| PEEP, cmH₂O | 1.053 (0.94–1.18) | 0.377 | 0.97 (0.82–1.16) | 0.753 |
| Driving pressure, cmH₂O | 1.131 (1.04–1.23) | 0.003 | 1.06 (0.85–1.31) | 0.632 |
| Respiratory system compliance, mL/cmH₂O | 0.98 (0.96–1.00) | 0.026 | 1.00 (0.97–1.03) | 0.863 |
| Peak airway pressure, cmH₂O | 1.07 (1.02–1.13) | 0.006 | 1.06 (0.94–1.19) | 0.383 |
| **Calculated P_mus_ subgroup** |  |  |  |  |
| 5–10 cmH₂O | Reference | — | Reference | — |
| <5 cmH₂O | 1.26 (0.56–2.81) | 0.577 | 0.88 (0.37–2.09) | 0.763 |
| >10 cmH₂O | 2.83 (1.20–6.66) | 0.018 | 2.61 (1.09–6.27) | 0.031 |

**Panel C. Calculated ΔP_L_ model**

| **Variable** | **Unadjusted HR (95% CI)** | ***P*** | **Adjusted HR (95% CI)** | ***P*** |
| --- | --- | --- | --- | --- |
| Tidal volume, mL | 1.00 (0.99–1.01) | 0.956 | 1.00 (1.00–1.01) | 0.615 |
| PEEP, cmH₂O | 1.05 (0.94–1.18) | 0.377 | 0.99 (0.82–1.19) | 0.901 |
| Driving pressure, cmH₂O | 1.13 (1.04–1.23) | 0.003 | 0.92 (0.73–1.17) | 0.490 |
| Respiratory system compliance, mL/cmH₂O | 0.98 (0.96–1.00) | 0.026 | 0.99 (0.96–1.03) | 0.611 |
| Peak airway pressure, cmH₂O | 1.07 (1.02–1.13) | 0.006 | 1.06 (0.93–1.19) | 0.397 |
| **Calculated ΔP_L_ subgroup** |  |  |  |  |
| ≤20 cmH₂O | Reference | — | Reference | — |
| >20 cmH₂O | 6.33 (3.02–13.29) | <0.001 | 6.00 (2.64–13.61) | <0.001 |

Abbreviations: HR, hazard ratio; PBW, predicted body weight; PEEP, positive end-expiratory pressure; Driving pressure; Pmus, calculated respiratory muscle pressure; ΔPL, calculated transpulmonary driving pressure.

Supplementary Table S4. Collinearity diagnostics for final multivariable models and physiology-driven sensitivity models

Panel A. Final multivariable models in the main analysis

| **Outcome / Variable** | **VIF** | **Tolerance** |
| --- | --- | --- |
| **28-day ventilator-free days** |  |  |
| Gender | 1.23 | 0.811 |
| Age | 1.13 | 0.886 |
| BMI | 1.27 | 0.787 |
| Interstitial lung disease | 1.12 | 0.889 |
| Immunosuppressive status | 1.17 | 0.852 |
| Malnutrition | 1.27 | 0.787 |
| Respiratory system compliance | 1.63 | 0.613 |
| Peak airway pressure | 2.38 | 0.421 |
| APACHE II score | 1.24 | 0.808 |
| P_0.1_ | 2.51 | 0.398 |
| Calculated P_mus_ | 3.63 | 0.275 |
| Calculated ΔP_L_ | 2.87 | 0.348 |
| **28-day mortality** |  |  |
| Age | 1.14 | 0.880 |
| APACHE II score | 1.17 | 0.852 |
| Immunocompromised status | 1.07 | 0.932 |
| Peak airway pressure | 2.03 | 0.493 |
| P_0.1_ | 2.56 | 0.391 |
| Calculated P_mus_ | 3.63 | 0.276 |
| Calculated ΔP_L_ | 3.00 | 0.334 |

Panel B. Physiology-driven sensitivity models

| **Outcome / Variable** | **VIF** | **Tolerance** |
| --- | --- | --- |
| Tidal volume, mL/kg PBW | 1.016 | 0.984 |
| PEEP, cmH₂O | 2.137 | 0.468 |
| Driving pressure, cmH₂O | 7.911 | 0.126 |
| Respiratory system compliance, mL/cmH₂O | 2.641 | 0.379 |
| Peak airway pressure | 6.849 | 0.146 |
| P_0.1_ | 2.526 | 0.396 |
| Calculated P_mus_ | 3.773 | 0.265 |
| Calculated ΔP_L_ | 3.121 | 0.320 |

**Footnote:** In the final main models, VIF values ranged from 1.07 to 3.63. In the physiology-driven sensitivity models, VIF values ranged from 1.016 to 7.911, with all tolerance values >0.10, supporting the absence of problematic multicollinearity in the retained models.

**Abbreviations:** BMI, body mass index;; APACHE II: Acute Physiology and Chronic Health Evaluation II, P_0.1_, airway occlusion pressure at 100 ms; P_occ_, occlusion pressure; P_mus_, calculated respiratory muscle pressure; ΔP_L_, calculated transpulmonary driving pressure.

**Supplementary Table S5. Cause-specific hazard of successful liberation from invasive mechanical ventilation by day 28**

| **Predictor** | **Comparison / unit** | **N** | **Events** | **HR (95% CI)** | ***P*** | **C-index** |
| --- | --- | --- | --- | --- | --- | --- |
| P_0.1_ during first 48 h | per unit increase | 206 | 149 | 1.03 (0.91–1.17) | 0.637 | 0.602 |
| Calculated P_mus_ during first 48 h | per unit increase | 206 | 149 | 1.02 (0.98–1.06) | 0.357 | 0.6027 |
| Calculated ΔP_L_ during first 48 h | per unit increase | 206 | 149 | 0.98 (0.95–1.01) | 0.106 | 0.6139 |
| RASS during first 48 h | per unit increase | 206 | 149 | 1.23 (1.09–1.39) | <0.001 | 0.6435 |
| P_0.1_ category | <1.5 vs 1.5–3.5 (reference) | 206 | 149 | 0.52 (0.37–0.73) | <0.001 | 0.6429 |
| P_0.1_ category | >3.5 vs 1.5–3.5 (reference) | 206 | 149 | 0.37 (0.16–0.86) | 0.021 | 0.6429 |
| Calculated P_mus_ category | <5 vs 5–10 (reference) | 206 | 149 | 0.66 (0.46–0.95) | 0.026 | 0.6208 |
| Calculated P_mus_ category | >10 vs 5–10 (reference) | 206 | 149 | 0.76 (0.50–1.17) | 0.210 | 0.6208 |
| Calculated ΔP_L_ >20 cmH2O | >20 vs ≤20 (reference) | 206 | 149 | 0.69 (0.49–0.98) | 0.037 | 0.6193 |
| Driving pressure | per unit increase | 206 | 149 | 0.96 (0.92–0.99) | 0.023 | 0.6168 |
| Peak airway pressure | per unit increase | 206 | 149 | 0.96 (0.94–0.99) | 0.003 | 0.6288 |
| Plateau pressure | per unit increase | 206 | 149 | 0.96 (0.93–0.99) | 0.005 | 0.6276 |
| Respiratory system compliance, per 10 mL/cmH_2_O | per 10 mL/cmH_2_O | 206 | 149 | 1.05 (1.01–1.08) | 0.012 | 0.6143 |

**Footnote:** In the cause-specific liberation model, successful liberation from invasive mechanical ventilation within 28 days was treated as the event of interest. Death before liberation was treated as a competing event, and patients alive but not liberated by day 28 were censored at day 28.

**Abbreviations:** HR, hazard ratio; CI, confidence interval; APACHE II, Acute Physiology and Chronic Health Evaluation II; P_0.1_, airway occlusion pressure at 100 milliseconds; P_mus_, calculated respiratory muscle pressure; ΔP_L_, calculated transpulmonary driving pressure; RASS, Richmond Agitation-Sedation Scale.

**Supplementary Table S6. Cause-specific hazard of death before liberation by day 28**

| **Predictor** | **Comparison / unit** | **N** | **Events** | **HR (95% CI)** | ***P*** | **C-index** |
| --- | --- | --- | --- | --- | --- | --- |
| P_0.1_ during first 48 h | per unit increase | 206 | 34 | 1.25 (1.04–1.51) | 0.016 | 0.6738 |
| Calculated P_mus_ during first 48 h | per unit increase | 206 | 34 | 1.08 (1.01–1.16) | 0.023 | 0.6923 |
| Calculated ΔP_L_ during first 48 h | per unit increase | 206 | 34 | 1.15 (1.09–1.22) | <0.001 | 0.7913 |
| RASS during first 48 h | per unit increase | 206 | 34 | 1.04 (0.79–1.37) | 0.773 | 0.6335 |
| P_0.1_ category | <1.5 vs 1.5–3.5 (reference) | 206 | 34 | 1.59 (0.62–4.07) | 0.332 | 0.7143 |
| P_0.1_ category | >3.5 vs 1.5–3.5 (reference) | 206 | 34 | 4.44 (1.51–13.04) | 0.007 | 0.7143 |
| Calculated P_mus_ category | <5 vs 5–10 (reference) | 206 | 34 | 0.72 (0.32–1.60) | 0.421 | 0.6692 |
| Calculated P_mus_ category | >10 vs 5–10 (reference) | 206 | 34 | 1.79 (0.77–4.20) | 0.178 | 0.6692 |
| Calculated ΔP_L_ >20 cmH2O | >20 vs ≤20 (reference) | 206 | 34 | 6.39 (2.93–13.91) | <0.001 | 0.7774 |
| Driving pressure | per unit increase | 206 | 34 | 1.12 (1.02–1.23) | 0.015 | 0.6812 |
| Peak airway pressure | per unit increase | 206 | 34 | 1.05 (0.99–1.10) | 0.087 | 0.6742 |
| Plateau pressure | per unit increase | 206 | 34 | 1.07 (1.00–1.15) | 0.052 | 0.6823 |
| Respiratory system compliance, per 10 mL/cmH_2_O | per 10 mL/cmH2O | 206 | 34 | 0.86 (0.63–1.17) | 0.337 | 0.6926 |

**Footnote:** In the cause-specific death model, death before successful liberation from invasive mechanical ventilation within 28 days was treated as the event of interest. Patients who were successfully liberated were censored at the time of liberation.

**Abbreviations:** HR, hazard ratio; CI, confidence interval; APACHE II, Acute Physiology and Chronic Health Evaluation II; P_0.1_, airway occlusion pressure at 100 milliseconds; P_mus_, calculated respiratory muscle pressure; ΔP_L_, calculated transpulmonary driving pressure; RASS, Richmond Agitation-Sedation Scale.

**Supplementary Table S7.** Spearman correlations between median P_0.1_ and other inspiratory drive/effort variables.

| Variable pair | n | Spearman r | ***P*** | 95%CI |
| --- | --- | --- | --- | --- |
| Median P_0.1_ vs median Pocc | 206 | 0.78 | <0.001 | 0.72-0.83 |
| Median P_0.1_ vs median calculated Pmus | 206 | 0.79 | <0.001 | 0.73-0.84 |
| Median P_0.1_ vs median calculated ΔPL | 206 | 0.30 | <0.001 | 0.17-0.43 |

**Abbreviations:** CI, confidence interval; P_0.1_, airway occlusion pressure at 100 ms; P_occ_, occlusion pressure; P_mus_, calculated respiratory muscle pressure; ΔP_L_, calculated transpulmonary driving pressure.
